# Perspectives of clinicians and survivors on the continuity of service provision during rehabilitation after acquired brain injury

**DOI:** 10.1371/journal.pone.0284375

**Published:** 2023-04-12

**Authors:** Rehab Alhasani, Dennis Radman, Claudine Auger, Anouk Lamontagne, Sara Ahmed

**Affiliations:** 1 School of Physical and Occupation Therapy, Faculty of Medicine, McGill University, Montreal, Canada; 2 Centre for Interdisciplinary Research in Rehabilitation of Greater Montreal (CRIR), Montreal, Quebec, Canada; 3 Department of Rehabilitation Sciences, College of Health and Rehabilitation Sciences, Princess Nourah Bint Abdulrahman University, Riyadh, Saudi Arabia; 4 School of Rehabilitation, Faculty of Medicine, Université de Montréal, Montreal, Quebec, Canada; 5 Institut universitaire sur la réadaptation en déficience physique de Montréal, CIUSSS du Centre-Sud-de-l’Île-de-Montréal, Montréal, Québec, Canada; 6 Jewish Rehabilitation Hospital, CISSS de Laval, Laval, Quebec, Canada; 7 Constance Lethbridge Rehabilitation Center, CIUSSS Centre- Ouest de l’Îile de Montreal, Montreal, Quebec, Canada; 8 McGill University Health Center Research Institute, Clinical Epidemiology, Center for Outcome Research and Evaluation (CORE), Montreal, Quebec, Canada; University of Ottawa, CANADA

## Abstract

The objective was to explore the care experiences and service design related to rehabilitation for mobility and participation in the community among individuals with acquired brain injury (ABI), as perceived by clinicians and patients. Five focus groups were held: three with clinicians and two with individuals with ABI. Focus group discussions were transcribed and analyzed using an inductive and deductive thematic content approach. Five themes were identified: Enabling continuity of care; System design; Accessibility and services in the community; Transportation services; and Uncertainty about the provided services. The results of participants’ experiences contributed to developing recommendations of service provision for mobility, leading to a patient-centered continuum of rehabilitation services. Accessibility to rehabilitation to improve the quality of care by addressing needs during transitions and mobility-related deficits, providing needed information, coordinated care, and self-management support in the community.

## Introduction

Acquired brain injury (ABI) including stroke and traumatic brain injury (TBI) is a significant cause of disability [1–4]. Approximately 1.5 million Canadians with ABI go through the acute and rehabilitation care continuum [5], costing the health system more than $26.8 billion annually [6]. Individuals with ABI face significant challenges, especially once discharged from acute care, in adjusting to a new phase of life, needing to manage expectations for recovery and potential functional independence [7, 8]. Mobility limitations are estimated to affect 30% of persons with TBI [1, 3, 4], and up to 50% of stroke survivors [9], even after extensive rehabilitation. Such mobility restrictions constrict community engagement and increase negative health outcomes and premature mortality [10, 11].

Mobility is a multidimensional construct with various operational definitions from theoretical and empirical approaches. From a theoretical point of view, some authors use an environmental continuum to define mobility as ‘life-space mobility [12–15]. Webber’s framework adds that mobility is influenced by five vital interrelated determinants, including physical, environmental, cognitive, psychosocial and financial [15]. Also, the broadness and complexity of all mobility-related domains is reflected in the International Classification, Functioning, Disability, and Health framework (ICF) mobility core set [16]. Furthermore, empirical studies based on the preceding frameworks showed that diagnosis alone is not enough to predict mobility limitations. For example, the length of hospitalization and intensity of care are needed to accurately predict a return to work potential, work performance, or social integration [17, 18]. Also, social and healthcare decision-makers recognize that decreasing the incidence and severity of disability and enhancing mobility and participation requires modifying features of the social and physical environment [16]. The World Health Organization recognized the necessity of an active participatory role for patients to improve both the quality of care and ease access to healthcare services [19].

While there have been significant efforts to optimise acute care and inpatient rehabilitation, there has been a lack of attention paid to long-term community care post-ABI [20, 21]. Once individuals with ABI are discharged from institutional care, many of them cannot access essential rehabilitation services such as physiotherapy, occupational therapy, and speech therapy [22]. Evidence has shown that the lack of community and primary care and services has led to a perception of marginalization and abandonment of ABI survivors and caregivers following inpatient discharge [23]. Systemic barriers to rehabilitation for individuals with ABI included a lack of a coordinated approach among healthcare and community service providers, difficulties in locating appropriate services, challenges in identifying professionals with ABI expertise, and an inability to find employment [20, 21]. There is a growing need for sufficient continuity from the time of rehabilitation admission to reintegration into the community, including availability of healthcare and community services that need to be accessed by individuals with ABI to improve mobility and participation into the community. The perspectives of clinicians and individuals with ABI are important to identify and develop solutions to gaps in health services.

### Objective

The objective was to explore the care experiences and service design related to rehabilitation for mobility and participation in the community among individuals with ABI, as perceived by clinicians and individuals with ABI.

## Materials and methods

### Statement of ethics

Approval of this study was granted by the Comité d’éthique de la recherche des établissements du centre de recherche interdisciplinaire en réadaptation (CRIR) [CRIR 1387–1218] on August 21, 2019.

### Research design, type of sampling and data collection

Focus group was chosen to facilitate discussions and exchange experiences of thoughts among a homogenous group of people related to a common topic [24, 25], and to produce a variety of ideas in a short time among participants [26, 27]. Data collection took place at three rehabilitation sites of Centre for Interdisciplinary Research in Rehabilitation of Greater Montreal (CRIR) in the province of Quebec, Canada. Pre-recruitment of individuals with ABI was accomplished using a computer-generated random list of previous rehabilitation clients in the sites since November 2019 using the following eligibility criteria: age ≥18 years; men or women with a primary diagnosis of stroke or TBI; files currently open or discharged six months; ability to speak French or English; and living in Montreal. First, based on a purposeful sampling strategy, a clinical team member called eligible participants to obtain initial verbal consent. Then a researcher contacted interested participants through phone calls, explained the study objectives, and answered questions.

Clinical research coordinators sent clinicians email invitations to participate, explaining the study’s objective. Interested clinicians contacted the researcher via email. Clinicians from different rehabilitation professions, with all years of experience working with individuals with ABI in inpatient, outpatient rehabilitation hospital settings, community care, or delivering home rehabilitation, and who spoke English or French were recruited based on a purposeful sampling strategy. All participants signed a consent form before attending the focus group discussions. Details of data collection and recruitment are presented elsewhere [28].

### Procedure

Five focus group discussions were conducted, three with clinicians at each rehabilitation site, one focus group discussion with individuals with stroke, and one virtual focus group discussion with individuals with TBI. A team of three clinical researchers (RA, SA, CA), reviewed the focus group interview guides, and iterative changes and reviews of all materials were completed to enhance the clarity of the documents (S1 Table).

Focus group discussions among clinicians and individuals with stroke were conducted in-person between November and December 2019, and lasted for two hours. For individuals with TBI, we used an online tool (Doodle), to find a common time across all participants with TBI for the focus group. Due to the current COVID-19 pandemic, we could not conduct the in-person focus group with individuals with TBI. Thus, an online focus group among individuals with TBI was held in May 2020. The group met virtually via a web video-conferencing platform (Zoom Video Communications Inc., 2020) for 90 minutes.

Participants connected to the meeting through Zoom via their computer, smartphone, or tablet, and joined using both video and audio. A key advantage of Zoom is its ability to securely record and store sessions without recourse to third-party software. This particular feature is essential in research where the protection of highly sensitive data is required. Other essential security features include user-specific authentication, real-time encryption of meetings, and the ability to backup recordings to online remote server networks (“the cloud”) or local drives, which can then be shared securely for the purpose of collaboration [29, 30]. The data from both formats (in-person and virtual) were combined and analyzed as one source [31]. After each focus group, a verbal summary was provided to participants to ensure clarity and accuracy of the content.

Two researchers (RA, SA) conducted the focus group discussions with open-ended questions, derived from the study objectives. Two co-moderators took notes during focus group discussions. An observer took additional notes and documented non-verbal communication. Pseudonyms were assigned to each participant. Quotes from French-speaking participants were translated into English. Focus group discussions were audio- recorded and transcribed verbatim after each session. To note, researchers, co-moderators, and observers were bilingual.

### Data analysis

Data were analysed using an inductive thematic content analysis, as described by Creswell [27]; and a deductive thematic content analysis using the ten rules for the ICF linking process [32] (S2 Table).

#### A) Data coding

In the first stage, the first author immersed herself in the data by repeatedly reading and listening to the recordings to become familiar with the data and document initial ideas arising from the audio and verbal material [27].

During the second stage, two independent reviewers (RA, DR) read each of the transcripts and line by line coding was undertaken independently using an open-ended approach to identify important concepts. Reviewers discussed and coded identified concepts. Codes were further discussed, and sub-themes were identified. These sub-themes were sorted, named, and organised into relevant themes considering the explicit aims of the study [27].

During the third stage, the ICF linking process, was used to analyze the data deductively [32] by the first author and then verified by the second author. The domains of each quote were linked to the ICF components of Body Functions, Activity and Participation and Contextual Factors. Domains were then linked at a general level (1-level classification) and expanded to levels of greater detail (2nd and 3rd specific ICF category) when the information was available. Resulting initial patterns were brought together, summarized, and refined. The two independent reviewers compared and debated their findings.

A third reviewer (SA) independently reviewed the provisional theme summaries from the second and third stages. Through iterative discussion and consultation during a series of virtual meetings among the reviewers (RA, DR, SA), themes were verified. Reviewers met regularly to resolve any discrepancies contributing to the consistency of the findings.

#### B) Code rating

In the second stage, the code rating was performed by calculating the frequency of each identified code corresponding to each theme among all participants. This process helped assess saturation based on the level of repetition of codes across all participants [33].

During the third stage, to identify the most prominent ICF domains, we mapped each code within each theme to the ICF domains. We then calculated the proportion of each code in each theme in relation to the ICF domains divided by the total number of codes in the theme.

### Triangulation, credibility and reflexivity

The primary means for ensuring trustworthiness was through triangulation, reflexivity, credibility, and peer debriefing. Triangulation of the data was achieved by conducting a focus group with individuals with ABI to corroborate or contrast with clinician perceptions [34]. Comparing notes and discussing expected and unexpected tangents between the focus group moderators, co-moderators, and observer throughout the data collection process facilitated reflexivity. After each focus group, a verbal summary was provided to participants to ensure the clarity and credibility of the data. Credibility [35], and trustworthiness [36] of data collection were ensured by cross-checking audio-files and transcripts by the reviewers, and the results were presented from all perspectives combined. Having multiple independent researchers code transcripts and compare codes through peer debriefing was a form of researcher triangulation and encouraged reflection on and refinement of categories as they were formulated [34].

## Results

### Participants’ characteristics

**Fig 1:**
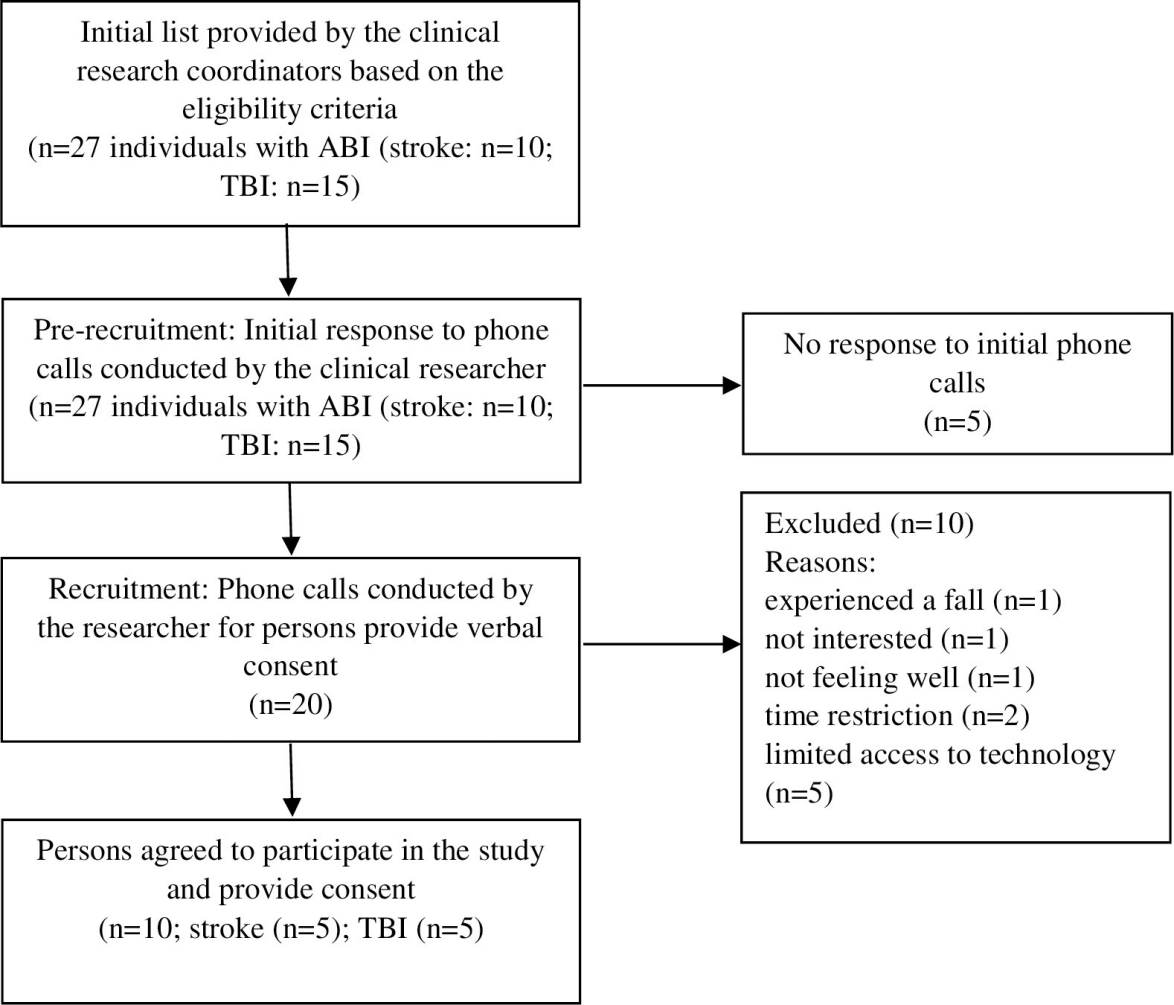
The recruitment process for individuals with acquired brain injury (ABI).

Seventeen clinicians from different professions were recruited and agreed to participate in the study. Three in-person focus groups were conducted, including 3 to 10 participants in each group. The fourth in-person focus group with individuals with stroke included five participants. The last focus group was conducted virtually among five female participants with TBI (Tables 1 & 2) [28]. The recruitment process for individuals with ABI is presented in Fig 1.

**Table 1:** Characteristics of clinicians.

Variables	Focus groups = 3; sample size = 17)
	n (%)
**Age (years)**	
**20–39**	6 (35)
**40–59**	11 (65)
**Age (M±SD) years**	41.35±10.28 years
**Sex**	
**Male**	1 (6)
**Female**	16 (94)
**Affiliated rehabilitation sites of CRIR**	
**CRDM**	4 (23)
**IURDPM**	3 (17)
**JRH**	10 (59)
**Profession**	
**Physiotherapists**	6 (35)
**Occupational therapists**	6 (35)
**Speech therapists**	1 (6)
**Psychologist**	2 (12)
**Social worker**	2 (12)
**Work position**	
**Full time/Permanent**	13 (76)
**Full time/Temporary**	1 (6)
**Part time/Permanent**	2 (12)
**Part time/Temporary**	1 (6)
**Work Settings**	
**Primary care**	2 (12)
**Secondary care**	10 (59)
**Tertiary care**	5 (29)
**Years of work experience (M±SD) years**	
**Practice (in general)**	15.79±8
**Practice with ABI**	11.56±6.78

CRIR: Centre for Interdisciplinary Research in Rehabilitation of Greater Montreal; CRDM: Constance Lethbridge Rehabilitation Center; IURDPM: Institut universitaire sur la réadaptation en déficience physique de Montréal; JRH:Jewish Rehabilitation Hospital.

**Table 2:** Characteristics of the individuals with acquired brain injury.

Variables	Focus group I (individuals with stroke; sample size = 5)	Focus group II (individuals with TBI; sample size = 5)
	n (%)	n (%)
**Age (years)**		
**20–39**	1 (20)	2 (40)
**40–59**	2 (40)	3 (60)
**60–79**	2 (20)	
**Age (M±SD) years**	58.4±15.69	43±17.24
**Sex**		
**Male**	4 (80)	
**Female**	1 (20)	5 (100)
**Affiliated rehabilitation sites of CRIR**		
**CRDM**	5 (100)	5 (100)
**IURDPM**		
**JRH**		
**Education**		
**Secondary school**	2 (40)	
**Bachelor degree**	3 (60)	5 (100)
**Marital status**		
**Married**	3 (60)	2 (40)
**Divorced**	1 (20)	1 (20)
**Single**	1 (20)	2 (40)
**Employment**		
**Full time worker**	1 (20)	2 (40)
**Part time worker**		1 (20)
**Unemployment**	2 (40)	
**Retired**	2 (40)	2 (40)
**Severity of injury**		
**Mild**	2 (40)	4 (80)
**Moderate**	3 (60)	1 (20)
**Severe**		
**Number of years living with ABI**		
**≤ 6 months**		1 (20)
**6 months-1 year**	1 (20)	2 (40)
**1 year-2 years**	4 (80)	2 (40)
**≥ 2 years**		
**Number of years (range)**	9 months-3 years	6 months- 2 years
**Type of focus group**	Face-to-face	Virtual-conferencing
**Type of technology used**		
**Iphone/ipad**	Not required	2 (40)
**Desktop**		2 (40)
**Laptop**		1 (20)

CRIR: Centre for Interdisciplinary Research in Rehabilitation of Greater Montreal; CRDM: Constance Lethbridge Rehabilitation Center; IURDPM: Institut universitaire sur la réadaptation en déficience physique de Montréal; JRH:Jewish Rehabilitation Hospital.

### Emerged themes

The emerged themes, and the number of codes for each theme by clinicians and individuals with ABI was presented in S2 Table.

### Theme 1: Enabling continuity of care

In healthcare contexts, continuity of care refers to care that occurs in consistent and coherent relationships with the patient about their care plan to sustain patient adherence to treatment goals [37, 38].

#### Clinicians’ perspectives

*1*.*1*. *Experiences with acute rehabilitation care*. Clinicians reported that they provide the needed education to the patient at the acute care setting to manage their symptoms and time to facilitate mobility and participation into the community (n = 2; 12%).

*C04: "we educate [clinicians working at acute setting] our patients with time management, because that has an impact into mobility, when to stop, when to start"*

*1*.*2*. *Transition from acute to rehabilitation settings*. Clinicians discussed many challenges that lead to substantial gaps between acute and rehabilitation care levels (n = 15; 88%). One of these challenges was related to workflow design which impacts clinical efficiency (n = 4; 23%). Another challenge was regarding young patients with impairments, as they usually get lost in the system after discharge from the acute care setting (n = 1; 6%). Clinicians suggested improving efficiency along the continuum of care, by increasing the speed of sharing discharge summaries from acute care settings using electronic health records instead of receiving paper summaries (n = 3; 17%). Using electronic health records can save time, and the patients can be admitted faster to outpatient rehabilitation.

*C03:" the thing to bring in the table is in getting the discharge summaries, I mean that is not even knowledge transfer that’s only information transfer"*

*C04:" the young clients are mostly lost in the system and get no service [when] they are the ones who they have the most potential to go back to work"*

*C03: "talking about having you know informatics electronic health records, so if it there it is saves time"*

A clinician discussed that outpatient waiting lists are long for patients that were discharged from the acute care setting, and they do not know how to solve the problem.

*C03:" we’ve got a huge waiting list"*

Clinicians expressed their uncertainty and difficulty to tailoring patient needs as the number of sessions is capped (n = 2; 12%). Prolonging services is not tenable due to the lack of resources.

*C03: "one of the challenges in outpatient rehab is we have to know when we stop treatment? And when they need to go to the next phase?"*

Clinicians discussed the factors that need to be considered when the patient is discharged from the acute care setting and has discharge planning (n = 9; 53%). Clinicians at acute care settings need to consider other deficits, and not just refer patients with mobility limitations to outpatient rehabilitation (n = 6; 35%). They explained that clinicians at acute care settings need to determine factors beyond the physical part of mobility, including safety perception, visual impairments, cognitive impairments, and aphasia impairments, and if they have a family member who can provide support once the patient is discharged. They highlighted that these factors would affect a patient’s mobility and be considered during transitions of care to ensure patients continue to receive the services needed. Clinicians described the importance of providing education and training for clinicians in acute care settings about not discharging patients based solely on their physical abilities (n = 1; 6%).

*C04: "if the [clients] had vision issues, vestibular issues, cognitive issues, or speech issues but physically they are fine, they will discharge with no service"*

*C06: "if the [patient] have a family support, to compensate, patients will be discharged faster, versus the person who is alone"*

*1*.*3*. *Access to rehabilitation setting in the community*. Clinicians pointed to the potential benefits of alternative secondary care services to persons with disability in the community while waiting for their admission to outpatient rehabilitation to facilitate mobility (n = 3; 17%). Due to limited resources in healthcare rehabilitation sectors, clinicians could not provide community services (n = 2; 12%).

*C05: "I find it’s the difficult area when our patients are discharged, when we know that he would have the potential to become independent but it’s as if the services don’t exist in a certain way or at least not in an optimal way to continue that with him"*

Clinicians discussed that some patients with stroke, who were discharged from the acute care setting, have access to Canadian Physiotherapy Association’s (CPA) programs and can get the needed care at their home (n = 2; 12%).

*C06: "Even for the stroke clientele, some patients don’t have access to the CPA program after discharge"*

*1*.*4*. *Reintegration into the community*. Clinicians reported challenges with patients applying what they have learned during outpatient rehabilitation sessions in the community (n = 4; 23%).

*C05: "we can do training [for people with cognitive impairments at the clinic] but they will have difficulty making the connections in their real environment"*

Clinicians cited the importance of having support services in the community that consider safety issues when assisting persons with ABI in becoming more functional and integrate into the community. Clinicians reported that safety should be considered before discharging patients to their home, especially for persons who live alone (n = 3; 17%).

*C06: "we try to put [the patients who are alone] in places [that offer] services to compensate for their safety"*

*1*.*5*. *Follow-up in the community*. Clinicians explained that the focus of the follow-up visits in the community varied depending on the site, ranging from mainly medical concerns to broad rehabilitation issues (n = 15; 88%). Clinicians were aware of the importance of adapting the visit to the participants’ home and often initiated the visit with a broad question "how are you doing?" or using a survey to facilitate the follow-up process (n = 1; 6%).

*C03: "if they got a survey monkey of something to say are there any problems, how are you doing? Would help"*

Clinicians in specialized care had a clear premise of the needs, and conducted a follow-up after 6 months from discharging individual with ABI from rehabilitation via phone calls (n = 6; 35%).

*C05: "[. . . .] we do follow-ups, which last between 6 months to sometimes 2 years"*

Clinicians proposed some ways to follow up and plan long-term by guiding the patients to be functional and safe (n = 8; 47%). Also, clinicians pointed out the limited resources to do the follow-up appropriately once patients were in the community (n = 2; 12%).

*C07: "We’re not the team that can do the training at their environment, [and the service] may take six months, we don’t have the Resources and service to train our patients at their home. I think that’s one of our big problems”*

Clinicians suggest some ways to follow up with their patients in the community using technology (n = 12; 70%). The follow-up would be facilitated by adapting tele-rehabilitation to be able to maintain the patient’s recovery. Some clinicians also described some challenges in using technology to follow up patients in the community, especially if they experienced cognitive impairments impacting their safety (n = 2; 12%).

*C01: "offering tele-rehab to those clients during winter time"*

*C05: "but for patients who are not completely safe, who is at risk of falling at home, tele-rehab would not work"*

Also, clinicians expressed challenges with following participants with TBI in the community. They added that they would be uncertain of how the patient was integrating into the community, especially if he had a cognitive impairment (n = 3; 17%).

*C05: "it’s over 60% of our entire clientele, older people with mild TBI and we have hard time following them up";"30% with moderate to severe TBI may be better to have them here than they leave to their home"*

#### Participants with ABI perspectives

*1*.*1*. *Experiences with acute rehabilitation care*. Participants with stroke expressed a feeling of mixed satisfaction with the rehabilitation services received in the acute setting. Some of them judged access to rehabilitation services to be easy in this type of setting because they were admitted to inpatient rehabilitation directly from emergency care (n = 2; 20%). Some participants experienced difficulty obtaining the services of a speech therapist or to have the opportunity to talk to their physician in the hospital setting (n = 6; 60%).

*S03: "the services I found the least helpful,[……] the speech therapy"*

Participants with stroke expressed their satisfaction with the quality of rehabilitation care services offered mainly by the physiotherapists and the occupational therapists (n = 4; 40%). However, some of them expressed how they felt very disappointed when the provider did not consider their reported deficits (such as cognition (n = 3; 30%)) and engage them as a whole person into their care (n = 5; 50%).

*S04: "I said why [it] seem to be having problems with my memory, I will go see my neurologist, and she [the speech therapist] said no neurologist can’t help you, she discouraged me"*

*S03: “[…] I found the physiotherapist very helpful and the occupational therapist over the speech therapy”*

*1*.*2*. *Transition from acute to rehabilitation settings*. Participants with stroke mentioned the importance to ease access to rehabilitation services to help with mobility and progress with recovery (n = 6; 60%); the wait to receive outpatient rehabilitation services after discharge from the acute setting was long (n = 3; 30%). This delay could affect the ability to improve their mobility. They were worried about their progress and claimed a reduction in wait times would fulfil their needs. A participant with stroke suggested that it is better to access private clinics, if possible, to continue to improve while waiting for outpatient rehabilitation services.

*S03: "Everybody wants to get better. Everybody wants access to; we need access to health services"; "as I probably speak mine, honestly unless you have like access to private"*

*S02:"the whole general system, it’s the waiting to get to [rehabilitation], so you don’t progress"*

*1*.*3*. *Reintegration into the community*. Participants with TBI reported that applying what they have learned into their life was challenging specifically when the provider did not provide the right guidance, support and honesty about the challenges and path of recovery that helped with re-integration into the community (n = 6; 60%).

*T01:"my first occupational therapist wasn’t really like telling me how to integrate myself really into regular life, like they were kind of just giving me some activities to do while I was there. And then we’d have a little talk but I didn’t like it I didn’t connect with them very much"*

Participants with stroke explained that some rehabilitation centres offered programs that were structured to simplify activities to facilitate mobility and reintegration into the community (n = 3; 30%).

*S04:"Like I remember one of the classes, they are talking occupation, like if you got to fold clothes, don’t stand over the table and do it, sit down and do it"*

### Theme 2: System design

System design defined as systematic changes to care practices and health systems to improve patient care quality, efficiency, and effectiveness [39].

#### Participants with ABI perspectives

*2*.*1*. *Quality of care*. Participants with TBI highlighted some difficulties when accessing specialized services (n = 6; 60%) and a perceived lack of expertise or knowledge from healthcare providers such as neurologists or general practitioners (n = 8; 80%). Participants with TBI report that some healthcare providers seemed to know little about TBI and its consequences and the TBI challenges of being an invisible disability. They could not provide the needed guidance. In contrast, they described that some healthcare providers know how to guide symptom management (n = 2; 20%).

*T01: I felt like the doctors that I saw didn’t really know, like they didn’t have very good suggestions of what to do and how to help"; "they definitely help [the healthcare providers] with like symptom management so symptom management was obviously like one of the biggest things"*

Participants with TBI and stroke reported not being consulted as much as they wished about important decisions such as therapeutic options or discharge (n = 4; 40%). They expressed a sense of emptiness after returning home and a feeling of being left behind.

*S03: "because she said there’s only so much she can do for me and I had to do it on my own [the speech therapist], I was discouraged actually to hear when she said that to me, I don’t know, I could have wanted more"*

*T02: "So we set some goals they never said after you finish those goals or you’re going to be finished"*

*S04: "I was there [at rehabilitation centre] for the stroke not for the pain right, so they didn’t take the pain into consideration at all"*

Participants with stroke who benefited from specialized rehabilitation care provided by some rehabilitation facilities claimed to be very satisfied with the expertise, the quality of care received, and the altruism of the care providers (n = 8; 80%).

*S04: "It’s good though, I found that the rehab centers are good, the doctors well good luck with that one! And then, it’s actually service that are missed still"*

*2*.*2*. *Information services*. Participants with ABI often found the information provided in the hospital setting to be limited, especially regarding their recovery prognosis, and to care planning and services to improve their mobility following discharge (n = 7; 70%). They expressed their needs in getting information services (n = 7; 70%), case management services (n = 5; 50%), to facilitate information exchange, ensure information retention, accountability (n = 2; 20%) and enhance patients’ navigation (n = 3; 30%). This need for information was directed toward outpatient rehabilitation services and highlighted their need to understand the evolution of their deficits and prevent further complications. Without information services, participants felt that they were left behind without guidance. They reported that they start to navigate the system by themselves and try to find a solution to prevent further complications (n = 3; 30%). Furthermore, participants with ABI mentioned that some clinicians provide honest and proper information to understand their deficits and plan for long-term solutions (n = 2; 20%).

*T01: "I don’t know, it’s kind of hard to navigate what to do because you didn’t really know who to listen to for where to turn"*

*S01: "where do I get that information or how do I get, you know this or like, anticipatory guidance"*

*T03: "my concussion was considered mild, but my therapist at xxx said sometimes there are a small percentage of cases with mild that just go on a very long time"*

*2*.*3*. *Oriented teamwork approach*. Participants with stroke found poor communication of salient information between healthcare providers, and they perceived the fragmentation of the information was greatest when some providers were stressed or overworked (n = 2; 20%).

*S03: "Some are really stressed out, in the health care system, they may start off with more ideal vision of what they like, and then in the end they end up being you know, overworked"*

*S04: "the Healthcare in Canada is great, but the disconnect with the doctor and the others is the problem"*

Participants with TBI explained a lack of coordination among healthcare providers and the absence of a teamwork-oriented culture (n = 7, 70%). This lack of coordination and education provision was misleading, leaving TBI survivors confused, and uncertain about their deficit and how to improve their mobility (n = 9; 90%).

*T03: "It was just not super contradicting but difference of opinions and you kind of don’t really know exactly what to listen to"*

Furthermore, participants with stroke and TBI reported that some healthcare providers might benefit from further training to improve the needed quality of care (n = 4; 40%).

*S06: "Proper training for the provider and teach them how to take care of the old man"*

*T01: "I think maybe like education [to the healthcare providers] about what exactly is going on and how they like see them like evolution like recovery"*

*2*.*4*. *Self-management*. Participants with TBI and stroke reported that the support they had anticipated was not available, be it a lack of therapy, lack of equipment, or difficulty arranging appointments with appropriate professionals. This makes it challenging to maintain therapy to bridge gaps while waiting to get the needed treatment in outpatient rehabilitation. For example, participants with ABI tend to use meta-cognitive strategies to progress their recovery and improve their mobility (n = 8; 80%).

*S03: "[the work] fell on me as well to make sure that I continue everything that I learned, you know and practicing it"; "and I really push through that to bridge my gap"*

*T02: "[self-management and writing] helped me to work with my symptoms like you know pacing, managing symptoms, and it also helps with the scheduling"*

### Theme 3: Accessibility and services in the community

#### Clinicians’ perspectives

Clinicians (n = 4; 23%) reported environmental barriers in the city that limit mobility; for example, there is no unique path for persons who are using a wheelchair. Also, with snow, there is a lack of services to keep pathways clean and accessible for persons with a disability, leading to social isolation and restricting their participation in the community. Also, they explained difficulties navigating areas of the city under construction, especially if the person has a cognitive impairment and uses a wheelchair at the same time. Clinicians (n = 4; 23%) reported that government policies related to disabilities and access to services did not meet patients’ needs.

*C02:"I find the city of Montreal, during the snowstorm, they don’t care if you are a handicap person, that’s it, and you are stuck inside"; " I think that the intervention doesn’t need to be with clients in itself, but with the city and the community"*

*C03: "construction is a big thing and navigating, especially if you have clients have cognition issue on top of that, they have hard time figuring out how to do it"*

#### Participants with ABI perspectives

Participants with stroke (n = 7; 70%) reported environmental barriers that limit mobility. For example, they could not use metros because of the lack of escalators. They reported that the environmental policies issued by the government did not consider disabilities needs.

*S01*: *"[in the metro station] there is no escalators right*, *so it’s like stairs*, *so how are you supposed to get down there*, *if like we do have a walker*, *you know*. *So*, *there’s a limitation*

"S04: "I think the laws about handicap service is disabled"

Participants with stroke (n = 6; 60%) claimed that they had difficulty obtaining assistance or care at home, especially when their problems were not physical. One participant reported that he adapted his home to make it more accessible, as he has a balance problem. Theses accessible design features included a shower bench, and bars in the house to enhance indoor mobility without using assistive devices.

*S05*:*"I have bars in my house*, *so I don’t use a cane inside; I got bars in the shower*, *now I don’t have to use a bench anymore"*

A participant with TBI reported that COVID19 impacted mobility and participation in the community, as the government closed the gym and recreational services.

*T02*: *"Well they close the gym [because of COVID]*. *So I had just started*, *I started just walking on the treadmill*. *I was a runner before so I can run"*

### Theme 4: Transportation services

#### Clinicians’ perspectives

Clinicians (n = 8; 47%) discussed the problems with adapted transport, as getting those services would typically take a long time. Also, adapted transport is not a good solution for persons with cognitive impairments, as it is difficult for them to manage or call for the service.

*C05*: *"the transport adapted is not easy to use for patients with too much cognitive impairment"; "it’s too difficult to manage*, *they’re not able to call*, *they’re not able to give all the information"*

Clinicians discussed some of the environmental limitations such as a snowstorm that make the transportation for persons with a disability difficult and limits their mobility, so they cannot get to their appointments at the outpatient rehabilitation setting (n = 3; 17%).

*C04*: *"if there is a snow storm*, *just forget it [because] the transportation is very late or transport adapted doesn’t park close to them"*

Clinicians discussed the importance of the availability of transportation services, as limits of public transportation services and limits of out-of-pocket access due to limited funds may limit mobility among individuals with ABI (n = 3; 17%).

*C06*: *"they don’t have the money to pay for a taxi every time*, *and transport adapted takes 2 months before they are accepted"*

#### Participants with ABI perspectives

Participants with stroke discussed how transportation services in the community were limited. They explained that the poor services were related to adapted transport services (n = 4; 40%), which accommodates for physical disability but not offered to those with cognitive limitations. There is a lack of transportation services for people with cognitive challenges, which impacts mobility. They also discussed challenges with adapting to using adapted transport services, which requires pre-planning, is more time-consuming, and can lead to being uncomfortable while in transport. The option of using other community transit services that offer help with mobility (buses, taxi) facilitates participation. However, the impact of noises and sensitivity to stimulation and tolerance to crowds limited their ability to use these services (n = 9; 90%). Participants discussed that some transportation services are beneficial as long as the people who provide the services have appropriate training (n = 2; 20%).

*S05*: *"I can do the buses for a decent amount now*, *as long as it’s not in rush hour or something"; "they drive you around for 2 hours*, *like crammed in the back of a hatchback with two other people*, *[while using transport adapted]"*

*S04*: "*Exactly, but the thing about the para transit is yeah you have this para transit service when you call and they are good, if they hire the taxis, and or they put them on the training course"*

Moreover, participants with stroke point out a lack of social norms (n = 5; 50%) for accessible seating on community transportations. They think that improving social norms on community transportation would help with mobility. Some people who provide public transportation were fighting for rights of persons with a disability and provide help to them, such as getting a seat on the bus.

*S05*:*"people really do not give us those handicap seats*, *if you have a cane they do not care*, *oh my goodness*, *I guess I’m too young or something"*

*S06*:"*I was taking the bus to the general [hospital] I think and I had my cane, when we got there, the bus driver stopped, looked up and said, hold on I will help you cross the street"; "that was exceptional, I was shocked, and I said, No, it’s okay, relax. But that’s one of how many?"*

### Theme 5: Uncertainty about the provided services

#### Participants with ABI perspectives

Participants with TBI reported that once discharged from formal rehabilitation care, provision for ABI was rationed, often leaving them with feelings of uncertainty regarding how best to manage their impairments and facilitate their mobility to participate better into the community (n = 5; 50%). Furthermore, in terms of whether or not people received information about the transition to community-based care concerning secondary prevention of ABI after discharge from either acute or rehabilitation level of care, the majority of participants initially reported having received little or no information when they were in hospital or rehabilitation, leaving them uncertain about how to progress or maintain recovery (n = 5; 50%). This disruption in rehabilitation services makes it hard for individuals with ABI to maintain progress.

*T01:"nobody can give you like a timeline or you’re never going to really be able to give a timeline, but more kind of an evolution symptoms, or maybe what to kind of expect based on your initial symptoms"*

Also, participants with TBI reported that the lack of transition services post-acute care and not getting the appropriate services because of the recent COVID19 pandemic impacted their recovery and mobility and left participants feeling uncertain about their progression (n = 5; 50%).

*T03:"But I just started my therapy when all this, when the COVID19 started so I really haven’t made any progress and I have no idea how to make progress at this point"*

#### The ICF linking process

All identified codes within all of the themes mapped to the ICF Environmental Factors. The exception was for theme 1, enabling continuity of care, where 80% (n = 100) mapped to the ICF Environmental Factors domain and the other 20% to Body Function (n = 6), Activity and Participation (n = 5), and not covered health conditions (n = 14) (S3 Table).

## Discussion

Experiences from the focus group discussions yielded an in-depth understanding of care experiences and service design related to rehabilitation for mobility and participation in the community among individuals with ABI. Through an in-depth inductive thematic analysis, five main themes, and nine subthemes emerged from the perceptions of clinicians and individuals with ABI. All themes align with the patient-centred care concept, including (1) Enabling continuity of care; (2) System design; (3) Accessibility and services in the community; (4) Transportation services; (5) Uncertainty about the provided services. Also, through a deductive thematic analysis using the ten-rule ICF linking process, most of the identified domains within each theme were mapped to the ICF Environmental Factors. The current study contributed clinicians’ and patients’ experience with service provision for mobility. Participants identified the need to address access to rehabilitation care, and specific areas needed to improve quality of care by addressing needs during transitions, addressing mobility-related deficits including cognition, vision, perceived safety, providing needed information, coordinated care, and self-management support in the community.

Combining inductive and deductive thematic analysis approaches allowed for a complete analysis and a critical realism ontological approach. While the inductive thematic analysis searched for patterns from raw data enabling the reality of others to be clearly represented, the deductive thematic analysis searched for consistencies and anomalies to provide an initial grounding of using a common language based on the ICF framework [40].

Generally, individuals with ABI experienced limited access to information related to recovery, prognosis, and to care planning following discharge from the acute setting. This included limited information about the available support services and resources in the community, therefore limiting their mobility and return to community activities. Additionally, participants with ABI had trouble accessing specialized services across the continuum of care, leading to feelings of abandonment. Overall, our results concur with recently published experiences of individuals with stroke with primary care and community healthcare services. Participants identified four essential services that were needed to improve quality of care: continuity of care, accessibility, information, and communication [23].

When considering rehabilitation care for mobility, individuals with ABI felt they needed information about their recovery and the rehabilitation process to enable them to make decisions related to their goals. Studies have shown that rehabilitation outcomes are improved when individuals with ABI actively participate in decision making and engage in their care [41]. Plant et al. [42] questioned whether individuals with ABI required directed goals during acute care given that some individuals expected to be guided by their providers, whereas in both rehabilitation and community settings, individuals were able to identify their goals and needs such that clinicians were able to embrace a patient-centred care process. Patients’ perceptions of clinician engagement in their care and the communication between patients and clinicians is essential in improving partnerships between patients and clinicians and influences individuals’ rehabilitation care experience [43].

Lack of engaging individuals with ABI, in rehabilitation decision processes may lead to disempowerment and may create a mismatch between individuals with ABI and healthcare provider expectations for recovery. A systematic review [44] reported that individuals with ABI more often chose goals to improve their level of participation in the community while most providers focused mainly on impairments and activity levels. Thus, engaging individuals in goal-setting and treatment planning and aligning patient and clinician expectations is important to achieve patient-centered care.

Our findings revealed that the level of knowledge and expertise of healthcare providers was considered to be an important factor in the quality of care across the continuum. Individuals with ABI felt that some healthcare providers were not knowledgeable enough to provide guidance and the needed care. Evidence in the literature showed that individuals with ABI prefer to receive their care from expert clinicians in a specialized care unit [45].

Moreover, results of this study showed that access to information was considered an essential concern among individuals with ABI across the continuum of care. They expressed wanting information on wait times for outpatient rehabilitation once discharged from acute care, expectations for recovery progress and expected improvements in mobility, and available community support services. ABI survivors reported their needs for more information on the availability of rehabilitation services and how to navigate access to services. Not getting the needed information can lead to fear, depression, and anxiety among individuals with ABI [46]. Additionally, it can hinder engaging the patient in their care as well as information exchange between patients and clinicians [47]. Not being equipped with information can also impede recovery progress and impact patients’ rehabilitation outcomes [46, 47].

Introducing the best way to provide information is not clear in the literature, but authors suggest using different active strategies that could engage patients in their care [48]. This could be done by providing education and counseling either face to face or through online technologies such as telehealth [48]. Studies have found that exchanging the needed information between clinicians and patients was essential for fostering a therapeutic alliance by sustaining trust and sharing power with patients [47]. Therefore, effective provision of information needs to consider the content, format, mode of delivery, and timing.

Limited access to specialized rehabilitation services once discharged from acute care settings was raised as an important barrier to recovery by individuals with ABI, and they felt services were non-existent in community settings. They needed to make substantial efforts to receive rehabilitation services when they were re-integrating into the community. Our results concur with results from published studies in the literature that have highlighted the difficulty to access rehabilitation services among individuals with ABI [20, 49].

Participants with ABI shared that it was difficult to access rehabilitation services when their mobility limitations were related to non-walking-related deficits. Evidence in the literature showed that most rehabilitation services offered to individuals with ABI concern mobility limitations; however, fewer rehabilitation services were offered to individuals with cognitive, speech, or visual impairments [49]. It is essential that individuals with impairments (such as cognition) limitations other than walking benefit from the rehabilitation services to ensure their safe integration into the community. Thus, raising awareness among the stakeholders about invisible disabilities and patients’ needs would result in better accessibility to rehabilitation services among individuals with ABI.

Results from this study indicated that there is a need to implement community education groups to better promote equitable rehabilitation services in the community. Community groups may create new social networks, and support learning, educational, and therapeutic opportunities among individuals with ABI [50]. Community-based therapy [51], such as group exercise [52] or aphasia therapy [52, 53] would support coping strategies and resilience and understanding of physical limitations as well as emotional cues. Thus, a collaboration between stakeholders would create a better therapeutic relationship between individuals with ABI and clinicians that could support an empowering environment.

A structured follow-up process initiated before discharge could improve access to healthcare services and help individuals with ABI identify solutions to address their needs. The problem of unequal follow-up was brought to light in this study and was represented by different prerequisites for specialized care in different settings. Problems discovered at a later stage, after discharge (such as cognitive impairments), indicate the need for long-term follow-up. In our study, clinicians in the rehabilitation setting were mainly satisfied with their current follow-up services; however, the follow-up only covered the first six months and did not provide continuity for longer-term support in the community. There is a lack of longer-term services that include holistic and coordinated support beyond the first six months [54]. Individuals’ preferences and needs, including previous experience with the available support services in the community, should be considered. In this way, recommendations and discharge rehabilitation goals and follow-up can be tailored to individuals’ physical and social context, and considers that individuals frequently prefer services they already know.

### Suggested solutions to make the continuity of care possible

Improving rehabilitation to enhance mobility will require increasing access to healthcare services across the continuum of care. Inpatient rehabilitation facilities often have limited access to specialized services [55]. Access to more comprehensive specialized care (e.g. speech therapy, cognitive retraining) in acute care is needed to address deficits early and minimize acute care transfers. One solution might be to embed an acute care hospital within the rehabilitation hospital to promote its capabilities. A triage room can allow for rapid evaluation and perhaps allow a return to the inpatient rehabilitation facilities, avoiding an acute care transfer [21, 23, 51, 55].

At the other end of the rehabilitation continuum, healthcare professionals and other stakeholders must engage patients, prepare them for discharge, and address their expectations. As there is increased demand for a shorter length of stay in hospitals, discharge planning must begin sooner [45, 55]. Support and training for the caregiver about the available resources in the community, and early evaluation of potential barriers to discharge are crucial. There is also a need for a multicenter study to examine whether specialized discharge programs for individuals with ABI, including individualized follow-up after discharge, can influence the transition to the community [45, 55].

Healthcare providers working in acute inpatient rehabilitation should ensure accurate and comprehensive information regarding the acute care stay. The involvement of a dedicated team member to provide systematic information, a psychologist or social worker, or peer navigator, in the acute care hospital, may facilitate information exchange [54, 55]. However, having a social worker or psychologist do this is expensive. It takes asking the patient questions, active listening, and filling in information gaps. A more realistic way is to have good quality educational material online that can also be printed to share with patients over time, adapting information to their stage of care. Also, a robust electronic health record system can assist in this process, which has the potential to help with information exchange, reduce practice differences, increase clinical productivity, and tailor care plans [55].

Using technology such as tele-rehabilitation to provide the needed care, education, and support to individuals with ABI outside the hospital is needed. Tele-rehabilitation allows long-term follow-up among individuals with ABI who may have difficulty with transportation, or are isolated by their disability, and need more efficient and timely access to their care. Using tele-rehabilitation is expected to result in a reduction of hospitalizations and lengths of hospital stay, and improve individuals’ quality of life [55]. Thus, tele-rehabilitation is one strategy that allows clinicians to assess and treat patients in different environments, especially in the community, exchanging the needed information and providing guidance to their clients [56].

Although telerehabilitation approaches have varied in the literature and have often been developed and evaluated with a limited theoretical basis [57], there are studies demonstrating results similar to traditional rehabilitation interventions. For example, providing an interactive and stimulating setting using a virtual reality approach can be achieved by 3-dimensional simulation that delivers a sense of engagement in a virtual environment that has been used in cognitive rehabilitation [58]. This could be enhanced by using, for example, robotic devices, data gloves, and smart glasses, that when combined with telerehabilitation technology would have the potential to enhance optimal rehabilitation care to improve patient mobility [58].

### Limitation

Findings of this qualitative study are based on a purposive sample and the study sample included more females than males. Therefore, results may not represent views of a broader population of clinicians working in a different setting, specifically in the community. Since most of the participants with ABI in the same focus group were recruited from one rehabilitation site, the results may not be generalizable. Hence, the results of this study should be interpreted cautiously. Future research may further distinguish the impact on caregiver experiences along the care continuum, contributing to the provision of timely support to improve health outcomes.

Although there is a great deal of interest in online focus group methods, less attention has been given to the quality of data they generate compared to the in-person focus group. Compared to the in-person focus group, the virtual focus group allowed participants to participate in a familiar environment instead of meeting in the same space [59]. This may reduce costs for researchers and participants, such as unnecessary travel. In the virtual one, it appeared that participants could express their opinions more comfortably. Moderators in an in-person focus group must work harder to control the flow of the discussion. Questioning, however, proved to be more difficult in the virtual focus group, as non-verbal or visual cues were more difficult to observe to allow the moderator to clarify further discussions [31, 59]. Although it is difficult to determine whether the differences occurred due to the focus group type, the findings suggest that the themes obtained from both formats were similar despite variations in word count per response. The results suggest that the role of the moderator in either setting was important for the data generated [31].

## Conclusion

The qualitative results of participants’ experiences contributed to developing recommendations of service provision for mobility leading to a patient-centred continuum of rehabilitation services from the acute level of care to community reintegration. Accessibility to rehabilitation care, and specific areas needed to improve quality of care by addressing needs during transitions and mobility related deficits (such as cognitive or aphasia impairments), providing needed information, coordinated care, and self-management support in the community. The results of this study can inform policy-makers, managers, administrators, clinicians, and researchers about services provision to improve mobility and participation in the community among individuals with ABI. The experiences can help identify the areas that need to be considered to develop ideal patient-centered rehabilitation services to improve individuals’ mobility and participation in life roles.

## Supporting information

S1 Table Focus group questions. (PDF)

S2 Table Thematic content analysis based on the coding rating (the frequency of each code within each theme among all participants). (PDF)

S3 Table Inductive and deductive thematic analysis. S3 Table legend C: clinician perspective; S: stroke perspective; T: traumatic brain injury perspective.
(PDF)

## References

[1] Mohamed Ludin S. and Rashid N.a. Abdul, Functional Outcomes and Health-Related Quality of Life After Severe Traumatic Brain Injury: A Review. Clinical Nursing Research, 2018. 10.1177/1054773818792459 30079766

[2] Ovbiagele B. and Nguyen-Huynh M.N., Stroke epidemiology: advancing our understanding of disease mechanism and therapy. Neurotherapeutics, 2011. 8(3): p. 319. 21691873 10.1007/s13311-011-0053-1 PMC3250269

[3] Ponsford J.L., et al., Longitudinal follow-up of patients with traumatic brain injury: Outcome at two, five, and ten years post-injury. Journal of Neurotrauma, 2014. 31: p. 64–77. 23889321 10.1089/neu.2013.2997

[4] Scholten A.C., et al., Health-related quality of life after mild, moderate and severe traumatic brain injury: Patterns and predictors of suboptimal functioning during the first year after injury. Injury, 2015. 46: p. 616–624. 25476014 10.1016/j.injury.2014.10.064

[5] Norris E., et al., Identifying and evaluating ontologies related to human behaviour change interventions: a scoping review. 2018.

[6] Parachute, The Cost of Injury in Canada [Internet]. Toronto, ON: Parachute, 2015.

[7] Han P., et al., Clinical evidence of exercise benefits for stroke, in Exercise for Cardiovascular Disease Prevention and Treatment. 2017, Springer. p. 131–151. 10.1007/978-981-10-4304-8_9 29098620

[8] Wein T., et al., Canadian stroke best practice recommendations: secondary prevention of stroke, practice guidelines, update 2017. International Journal of Stroke, 2018. 13(4): p. 420–443. 29171361 10.1177/1747493017743062

[9] Robinson C.A., et al., Understanding physical factors associated with participation in community ambulation following stroke. Disability and Rehabilitation, 2011. 33: p. 1033–1042. 20923316 10.3109/09638288.2010.520803

[10] Carroll L., et al., Prognosis for mild traumatic brain injury: results of the WHO Collaborating Centre Task Force on Mild Traumatic Brain Injury. Journal of rehabilitation medicine, 2004. 36(0): p. 84–105. 10.1080/16501960410023859 15083873

[11] Hole E., et al., The patient’s experience of the psychosocial process that influences identity following stroke rehabilitation: a metaethnography. The Scientific World Journal, 2014. 2014. 10.1155/2014/349151 PMC3927748 24616623

[12] Shumway-Cook A., et al., Environmental demands associated with community mobility in older adults with and without mobility disabilities. Physical therapy, 2002. 82(7): p. 670–681. 12088464

[13] Tsai L.-T., Walking, physical activity and life-space mobility among older people. Studies in sport, physical education and health, 2017(254).

[14] Peel C., et al., Assessing mobility in older adults: the UAB Study of Aging Life-Space Assessment. Physical therapy, 2005. 85(10): p. 1008–1019. 16180950

[15] Webber S.C., Porter M.M., and Menec V.H., Mobility in older adults: A comprehensive framework. Gerontologist, 2010. 50: p. 443–450. 20145017 10.1093/geront/gnq013

[16] Organization, W.H., International classification of functioning, disability and health: ICF. 2001: Geneva: World Health Organization.

[17] Reichard A., et al., Diagnosis isn’t enough: understanding the connections between high health care utilization, chronic conditions and disabilities among US working age adults. Disability and health journal, 2015. 8(4): p. 535–546. 26082321 10.1016/j.dhjo.2015.04.006 PMC4570849

[18] Wise F.M., et al., Acute predictors of social integration following mild stroke. Journal of stroke and Cerebrovascular Diseases, 2018. 27(4): p. 1025–1032. 29249589 10.1016/j.jstrokecerebrovasdis.2017.11.011

[19] Europe, W.R.O.f., What is the Evidence on Existing Policies and Linked Activities and Their Effectiveness for Improving Health Literacy at National Regional and Organizational Levels in the WHO European Region? Vol. 57. 2018: World Health Organization. 30230797

[20] Borg D.N., et al., The effect of access to a designated interdisciplinary post-acute rehabilitation service on participant outcomes after brain injury. Brain injury, 2020. 34(10): p. 1358–1366. 32780595 10.1080/02699052.2020.1802660

[21] Harrison A.L., et al., Living with traumatic brain injury in a rural setting: supports and barriers across the continuum of care. Disability and rehabilitation, 2017. 39(20): p. 2071–2080. 27549899 10.1080/09638288.2016.1217081 PMC5654530

[22] Hartford W., Lear S., and Nimmon L., Stroke survivors’ experiences of team support along their recovery continuum. BMC health services research, 2019. 19(1): p. 1–12. 31638959 10.1186/s12913-019-4533-z PMC6805495

[23] Pindus D.M., et al., Stroke survivors’ and informal caregivers’ experiences of primary care and community healthcare services–a systematic review and meta-ethnography. PLoS One, 2018. 13(2): p. e0192533. 29466383 10.1371/journal.pone.0192533 PMC5821463

[24] Kinalski D.D.F., et al., Focus group on qualitative research: experience report. Revista Brasileira de Enfermagem, 2017. 70(2): p. 424–429. 28403311 10.1590/0034-7167-2016-0091

[25] Morgan D.L. and Krueger R.A., Analyzing and reporting focus group results. 1998: Sage.

[26] Carpenter C., Using qualitative focus groups to evaluate health programmes and service delivery, in Qualitative research in evidence-based rehabilitation. 2004, Elsevier. p. 51–64.

[27] Creswell J.W., Qualitative inquiry & research design: choosing among five approaches. 2007: p. 395.

[28] Alhasani R., et al., Clinicians and individuals with acquired brain injury perspectives about factors that influence mobility: creating a core set of mobility domains among individuals with acquired brain injury. Annals of medicine, 2021. 53(1): p. 2365–2379. 34894914 10.1080/07853890.2021.2015539 PMC8676689

[29] Archibald M.M., et al., Using Zoom Videoconferencing for Qualitative Data Collection: Perceptions and Experiences of Researchers and Participants. International Journal of Qualitative Methods, 2019. 18.

[30] Ting D.S.W., et al., Digital technology and COVID-19, in Nature Medicine. 2020, Nature Research. p. 459–461. 10.1038/s41591-020-0824-5 PMC7100489 32284618

[31] Woodyatt C.R., Finneran C.A., and Stephenson R., In-person versus online focus group discussions: A comparative analysis of data quality. Qualitative Health Research, 2016. 26(6): p. 741–749. 26935719 10.1177/1049732316631510

[32] Cieza A., et al., Refinements of the ICF Linking Rules to strengthen their potential for establishing comparability of health information. Disability and rehabilitation, 2019. 41(5): p. 574–583. 26984720 10.3109/09638288.2016.1145258

[33] Bernard H.R., Research methods in anthropology: Qualitative and quantitative approaches. 2017: Rowman & Littlefield.

[34] Carter N., et al., The use of triangulation in qualitative research, in Oncology Nursing Forum. 2014, Oncology Nursing Society. p. 545–547. 10.1188/14.ONF.545-547 25158659

[35] Hayashi P., Abib G., and Hoppen N. Validity in Qualitative Research: A Processual Approach. in The Qualitative Report. 2019. 10.1177/00469580211060750 PMC8640329 34845941

[36] Wood L.M., Sebar B., and Vecchio N. Application of Rigour and Credibility in Qualitative Document Analysis: Lessons Learnt from a Case Study. in The Qualitative Report. 2020.

[37] Ruben M.A., Blanch‐Hartigan D., and Hall J.A., Patients in Treatment. The Wiley Handbook of Healthcare Treatment Engagement: Theory, Research, and Clinical Practice, 2020: p. 274.

[38] Wang D., Liu C., and Zhang X., Do Physicians’ Attitudes towards Patient-Centered Communication Promote Physicians’ Intention and Behavior of Involving Patients in Medical Decisions? International journal of environmental research and public health, 2020. 17(17): p. 6393. 32887364 10.3390/ijerph17176393 PMC7503802

[39] Dabney B.W. and Tzeng H.-M., Service quality and patient-centered care. Medsurg Nursing, 2013. 22(6). 24600931

[40] Boyatzis R.E., Transforming qualitative information: Thematic analysis and code development. 1998: sage.

[41] Lawrence M. and Kinn S., Defining and measuring patient‐centred care: an example from a mixed‐methods systematic review of the stroke literature. Health Expectations, 2012. 15(3): p. 295–326. 21624025 10.1111/j.1369-7625.2011.00683.x PMC5060626

[42] Plant S.E., et al., What are the barriers and facilitators to goal-setting during rehabilitation for stroke and other acquired brain injuries? A systematic review and meta-synthesis. Clinical rehabilitation, 2016. 30(9): p. 921–930. 27496701 10.1177/0269215516655856 PMC4978164

[43] Bright F.A., et al., Co-constructing engagement in stroke rehabilitation: a qualitative study exploring how practitioner engagement can influence patient engagement. Clinical rehabilitation, 2017. 31(10): p. 1396–1405. 28653548 10.1177/0269215517694678 PMC5613802

[44] Rosewilliam S., Roskell C.A., and Pandyan A., A systematic review and synthesis of the quantitative and qualitative evidence behind patient-centred goal setting in stroke rehabilitation. Clinical rehabilitation, 2011. 25(6): p. 501–514. 21441308 10.1177/0269215510394467

[45] Sugavanam T., et al., The effects and experiences of goal setting in stroke rehabilitation–a systematic review. Disability and rehabilitation, 2013. 35(3): p. 177–190. 22671934 10.3109/09638288.2012.690501

[46] Morris R., Payne O., and Lambert A., Patient, carer and staff experience of a hospital-based stroke service. International Journal for Quality in Health Care, 2007. 19(2): p. 105–112. 17277009 10.1093/intqhc/mzl073

[47] Clarke S., et al., Defining elements of patient-centered care for therapeutic relationships: A literature review of common themes. European journal for person centered healthcare, 2017. 5(3): p. 362–372.

[48] Smith J., et al., Cochrane review: information provision for stroke patients and their caregivers. Clinical rehabilitation, 2009. 23(3): p. 195–206. 19218295 10.1177/0269215508092820

[49] Vincent C., et al., Provision of rehabilitation services in Québec following stroke: a comparative survey conducted by postal questionnaire. Canadian Journal on Aging/La revue canadienne du vieillissement, 2010. 29(2): p. 193–203. 20465861 10.1017/S0714980810000127

[50] Hou S.-I., Health education: theoretical concepts, effective strategies and core competencies. Health promotion practice, 2014. 15(5): p. 619–621.

[51] Graven C., et al., Stroke survivor and carer perspectives of the concept of recovery: a qualitative study. Disability and rehabilitation, 2013. 35(7): p. 578–585. 22889405 10.3109/09638288.2012.703755

[52] Mayo N.E., et al., Getting on with the rest of your life following stroke: a randomized trial of a complex intervention aimed at enhancing life participation post stroke. Clinical rehabilitation, 2015. 29(12): p. 1198–1211. 25627292 10.1177/0269215514565396

[53] Gallacher K., et al., Uncovering treatment burden as a key concept for stroke care: a systematic review of qualitative research. PLoS Med, 2013. 10(6): p. e1001473. 23824703 10.1371/journal.pmed.1001473 PMC3692487

[54] Kjörk E.K., et al., Experiences, needs, and preferences for follow-up after stroke perceived by people with stroke and healthcare professionals: A focus group study. PloS one, 2019. 14(10): p. e0223338. 31574135 10.1371/journal.pone.0223338 PMC6772122

[55] Watanabe T.K., Esquenazi A., and Flanagan S., The transformation of the rehabilitation paradigm across the continuum of care. PM&R, 2018. 10(9): p. S264–S271. 30269811 10.1016/j.pmrj.2018.08.381

[56] Parmanto B. and Saptono A., Telerehabilitation: State-of-the-art from an informatics perspective. International Journal of Telerehabilitation, 2009. 1(1): p. 73. doi: 10.5195/ijt.2009.6015 25945164 PMC4296781

[57] Peretti A., et al., Telerehabilitation: Review of the State-of-the-Art and Areas of Application. JMIR rehabilitation and assistive technologies, 2017. 4(2): p. e7. doi: 10.2196/rehab.7511 28733271 PMC5544892

[58] Chen J., et al., Telerehabilitation approaches for stroke patients: systematic review and meta-analysis of randomized controlled trials. Journal of Stroke and Cerebrovascular Diseases, 2015. 24(12): p. 2660–2668. 26483155 10.1016/j.jstrokecerebrovasdis.2015.09.014

[59] Stewart D.W. and Shamdasani P., Online focus groups. Journal of Advertising, 2017. 46(1): p. 48–60.

