# Supplementary material for: Perspectives of clinicians and survivors on the continuity of service provision during rehabilitation after acquired brain injury
Source: PLoS One. 2023 Apr 12;18(4):e0284375. doi: 10.1371/journal.pone.0284375 (PMC10096466; doi:10.1371/journal.pone.0284375)
Supplement: S1 Table — (PDF) [file pone.0284375.s001.pdf]

**S1 Table. Focus group questions**

| | |
|---|---|
| **Clinicians guiding questions** | |
| 1) | What are the questions that you have in relation to your practice delivering rehabilitation to individuals with acquired brain injury (ABI)? |
| 2) | In general, what are the areas that you would like to improve either in your individual practice or rehabilitation among individuals with ABI? |
| 3) | How do you define mobility? [The focus group members will agree on a definition on mobility that will be read back to the focus group participants] |
| 4) | What are the important factors that you believe influence mobility? |
| 5) | What do you feel you need to evaluate to have a good picture of a person's mobility while the person with ABI in rehabilitation (inpatient/outpatient)? |
| 6) | What do you feel you need to evaluate to have a good picture of a person's mobility while the person with ABI in community? |
| 7) | From the inventory, how did you choose these measures? Is it capturing all aspects of mobility? |
| 8) | What is your perception in regards to mobility measures that were captured from the literature and were not proposed in the inventory? |
| 9) | Consider mobility in rehabilitation setting; what do you see are the challenges of using outcome measures in this environment and what can be done to make it easier to use them? |
| 10) | Now consider mobility in the community; What do you see are the challenges of using outcome measures in this environment and what can be done to make it easier to use them? |
| 11) | From your experience regarding the use of mobility measures, how do you use the scores to guide the development of the intervention plan? |
| **Individuals with acquired brain injury and their caregivers guiding questions** | |
| 1) | As an individual with stroke or brain injury, what are the questions that you have in relation to your condition and to care you received since you have your incidence. (This can include care at the hospital, rehabilitation, or with community care providers including your family doctor)? |
| 2) | What are the areas that you would like to improve in rehabilitation health care system (if any) to get better care? |
| 3) | What has been your experience in terms of your daily activities, including work or school, or in participating in social activities with family and friends? |
| 4) | Were you involved as much as you wanted to be in decisions about your care and treatment? |
| 5) | How do you define mobility? [The focus group members will discuss a definition of mobility that will be read back to the focus group participants for further feedback] |
| 6) | What are the important factors (e.g, cognition, and environment) that you believe influence mobility? |
| 7) | As someone lives with stroke or brain injury and/or a caregiver supporting someone with stroke or brain injury, what do you feel needs to be measured or monitored in relation to mobility while someone is in the hospital? |
| 8) | As someone lives with stroke or brain injury and/or a caregiver supporting someone with stroke or brain injury, what do you feel needs to be measured or monitored in relation to mobility in community? |
| 9) | Please explain how rehabilitation care prepared you to return home/ back to your work (if relevant)/ school (if relevant), and community? |
| 10) | Consider mobility in rehabilitation setting; what are the challenges that you face in this environment and what can be done to overcome these challenges? |
| 11) | Now consider mobility in the community; what are the challenges that you face in this environment and what can be done to overcome these challenges? |
