# Supplementary material for: Perspectives of clinicians and survivors on the continuity of service provision during rehabilitation after acquired brain injury
Source: PLoS One. 2023 Apr 12;18(4):e0284375. doi: 10.1371/journal.pone.0284375 (PMC10096466; doi:10.1371/journal.pone.0284375)
Supplement: S2 Table — (PDF) [file pone.0284375.s002.pdf]

S2 Table. Thematic content analysis based on the coding rating (the frequency of each code within each theme among all participants)

Codes	All participants (n=28) n (%)	Clinicians (n=17) n (%)	Patients (n=10) n (%)
**Theme 1: Enabling continuity of care**			
**1.1. Experiences with acute rehabilitation care**			
• Quality of services	7 (25%)	2 (12%)	4 (40%)
• Individualized focus	6 (21%)		5 (50%)
• Specialized Services	6 (21%)		6 (60%)
• Accessibility to healthcare services	3 (11%)		2 (20%)
• Deficits	3 (11%)		3 (30%)
• Beside manner	3 (11%)		3 (30%)
• Education services to patients	2 (7%)	2 (12%)	
• Language barrier	1 (3%)		
**1.2. Transition from acute to rehabilitation settings**			
• Service Pathways	15 (53%)	15 (88%)	
• Discharge factors	9 (32%)	9 (53%)	
• Accessibility to healthcare services	7 (25%)		6 (60%)
• Deficits	6 (21%)	6 (35%)	
• Wait times	5 (18%)	1 (6%)	3 (30%)
• Workflow design	4 (14%)	4 (23%)	
• Specialized services	4 (14%)		3 (30%)
• Electronic health records	3 (11%)	3 (17%)	
• Uncertainty	2 (7%)	2 (12%)	
• Quality of services	2 (7%)		1 (10%)
• Young patients with impairments	1 (3%)	1 (6%)	
• Education services to providers	1 (3%)	1 (6%)	
• Caregiver support services	1 (3%)		
• Information services	1 (3%)		
• Family Support	1 (3%)	1 (6%)	
**1.3. Access to rehabilitation in the community setting**			
• Specialized services	4 (14%)	3 (17%)	1 (10%)
• Deficits	3 (11%)	3 (17%)	
• Discharge factors	3 (11%)	3 (17%)	
• Accessibility to healthcare services	3 (11%)	2 (12%)	1 (10%)
• Resources	2 (7%)	2 (12%)	
**1.4. Re-integration into the community**			
• Re-integration	10 (36%)	4 (23%)	6 (60%)
• Education services to patients	3 (11%)		3 (30%)
• Individualized focus	3 (11%)		3 (30%)
• Support services	3 (11%)	3 (17%)	
• Safety	3 (11%)	3 (17%)	
• Specialized services	3 (11%)		3 (30%)
• Deficits	1 (3%)	1 (6%)	
**1.5. Follow-up in the community**			
• Follow-up	15 (53%)	15 (88%)	

Codes	All participants (n=28) n (%)	Clinicians (n=17) n (%)	Patients (n=10) n (%)
• Technology/Tele-health	12 (42%)	12 (70%)	
• Safety	8 (28%)	8 (47%)	
• Support services	6 (21%)	6 (35%)	
• Deficits	3 (11%)	3 (17%)	
• Resources	2 (7%)	2 (12%)	
• Survey	1 (3%)	1 (6%)	
**Theme 2: System Design**			
**2.1. Quality of care**			
• Quality of services	10 (36%)	1 (6%)	8 (80%)
• Knowledge acquisition	8 (28%)		8 (80%)
• Specialized therapy	6 (21%)		6 (60%)
• Healthcare services	5 (18%)	1 (6%)	3 (30%)
• Individualized focus	4 (14%)		4 (40%)
• Symptom management	2 (7%)		2 (20%)
**2.2. Information services**			
• Information services	7 (25%)		7 (70%)
• Case management services	5 (18%)		5 (50%)
• Navigate system	3 (11%)		3 (30%)
• Accountability	2 (7%)		2 (20%)
**2.3. Oriented teamwork approach**			
• Quality of services	9 (32%)		9 (90%)
• Team-oriented	7 (25%)		7 (70%)
• Education to providers	4 (14%)		4 (40%)
• Overworked professionals	2 (7%)		2 (20%)
**2.4. Self-management**			
• Self-therapy	8 (28%)		8 (80%)
**Theme 3: Accessibility and system in the community**			
• Policy	11 (39%)	4 (23%)	7 (70%)
• Accessibility design	10 (36%)	4 (23%)	6 (60%)
• Recreational services	1 (3%)		1 (10%)
**Theme 4: Transportation services**			
• Transport adapted services	13 (46%)	8 (47%)	4 (40%)
• Public transportation services	10 (36%)	1 (6%)	9 (90%)
• Social norms	5 (18%)		5 (50%)
• Weather	3 (11%)	3 (17%)	
• Training	2 (7%)		2 (20%)
• Financial limitation	1 (3%)	1 (6%)	
**Theme 5: Uncertainty about the provided services**			
• Uncertainty	5 (18%)		5 (50%)
