# Supplementary material for: Perspectives of clinicians and survivors on the continuity of service provision during rehabilitation after acquired brain injury
Source: PLoS One. 2023 Apr 12;18(4):e0284375. doi: 10.1371/journal.pone.0284375 (PMC10096466; doi:10.1371/journal.pone.0284375)
Supplement: S3 Table — legend C: clinician perspective; S: stroke perspective; T: traumatic brain injury perspective. (PDF) [file pone.0284375.s003.pdf]

# S3 Table. Inductive and deductive thematic analysis

| Quotes | Population | Formulated statements | Codes | ICF domain | 1st Level ICF classification | 2nd Level ICF category | 3rd or higher Level ICF category |
|---|---|---|---|---|---|---|---|
| **Theme 1: Enabling continuity of care** | | | | | | | |
| **1.1. Experiences with acute rehabilitation care** | | | | | | | |
| *S03: "the services I found the least helpful, I found the physiotherapist very helpful and the occupational therapist over the speech therapy"* | Stroke | Physiotherapy and occupational therapy helpful and better than speech therapy | Specialized Services | Environmental Factor | e5 Services, systems and policies | e580 Health services, systems and policies | e5808 Health services, systems and policies, other specified (Specialized services) |
| *S02: "I was taken by ambulance to the xxx and you're right the service there is like amazing and then that night I got transferred to the neuro because they didn't have a bed for me at the general which is also kind of a blessing in disguise"* | Stroke | Quality of services at some hospitals was excellent | Accessibility to healthcare<br>Quality of services | Environmental Factor | e5 Services, systems and policies | e580 Health services, systems and policies | e5809 Health services, systems and policies, other unspecified |
| *S03: "the service I found at this hospital xxx was the least helpful"* | Stroke | Some services don't help | Specialized Services | Environmental Factor | e5 Services, systems and policies | e580 Health services, systems and policies | e5808 Health services, systems and policies, other specified (Specialized services) |
| *S03: "Sometimes it doesn't connect. Exactly she just was not just my thing [the occupational therapist]"* | Stroke | Occupational therapy not helpful | Specialized Services | Environmental Factor | e5 Services, systems and policies | e580 Health services, systems and policies | e5808 Health services, systems and policies, other specified (Specialized services) |
| *S04: "Others are terrible. Starting off with the doctor, you go see neurologist for 5 or 10 minutes while he's on the phone talking that's the first problem, they [the neurologist] don't care"* | Stroke | Physicians don't help | Specialized Services | Environmental Factor | e5 Services, systems and policies | e580 Health services, systems and policies | e5808 Health services, systems and policies, other specified (Specialized services) |
| *S05: "I wasn't able to work full time before the stroke, I had a serious injury, but 10 minutes with the neurologist, after the stroke, Oh now you're good to work full time!"* | Stroke | Physicians don't help/ Beside manners | Specialized Services<br>Accessibility to healthcare services<br>Beside manners | Environmental Factor | e5 Services, systems and policies | e580 Health services, systems and policies | e5808 Health services, systems and policies, other specified (Specialized services) |
| *S04: "I said why seem to be having problems with my memory, I will go see my neurologist, and she [the speech therapist] said no neurologist can't help you, she discouraged me"* | Stroke | Speech therapy not helpful/ Respecting patients needs and preferences/ Memory problem | Specialized Services<br>Individualized focus<br>Deficits | Environmental Factor<br><br>Body Function | e5 Services, systems and policies<br>b1 Mental Functions | e580 Health services, systems and policies<br>b144 Memory functions | e5808 Health services, systems and policies, other specified (Specialized services)<br>b1449 Memory functions, unspecified |

| Quotes | Population | Formulated statements | Codes | ICF domain | 1st Level ICF classification | 2nd Level ICF category | 3rd or higher Level ICF category |
|---|---|---|---|---|---|---|---|
| *S04: "I couldn't care less, so I contact neurologist, and consequently maybe it's not really related to strokes"* | Stroke | Decisions made by patients without consulting the healthcare provider | Individualized focus | Environmental Factor | e5 Services, systems and policies | e580 Health services, systems and policies | e5809 Health services, systems and policies, other unspecified |
| *S04: "the OT there she noticed, and gave you a quick memory test, and I got she goes wow!, you do have a memory problem"* | Stroke | Occupational therapy was helpful/ Memory problem | Individualized focus<br>Deficits | Environmental Factor<br>Body Function | e5 Services, systems and policies<br>b1 Mental Functions | e580 Health services, systems and policies<br>b144 Memory functions | e5808 Health services, systems and policies, other specified (Specialized services)<br>b1449 Memory functions, unspecified |
| *S04: "because of her [the OT], getting hooked up with a neuropsychologist that does like 12 hours of testing and they found stuff"* | Stroke | Occupational therapy was helpful/ Memory problem | Individualized focus<br>Deficits | Environmental Factor<br><br>Body Function | e5 Services, systems and policies<br>b1 Mental Functions | e580 Health services, systems and policies<br>b144 Memory functions | e5808 Health services, systems and policies, other specified (Specialized services) |
| *T03:"So it's just having people believe you and support you and understand the difficulties that you're going through finding those health professionals, especially at the neuroscientist kind of level "* | TBI | Support is important, listening, understanding is important | Quality of services | Environmental Factor | e5 Services, systems and policies | e580 Health services, systems and policies | e5809 Health services, systems and policies, other unspecified |
| *T03:"Who You would think would be extremely helpful, but I found that neuro physician. The most dismissive of all"* | TBI | Neurologist is dismissive; bedside manner is poor | Quality of services<br>Beside manner | Environmental Factor | e5 Services, systems and policies | e580 Health services, systems and policies | e5809 Health services, systems and policies, other unspecified |
| *T04:"I found a lot of they [the doctors] weren't taking me seriously"* | TBI | Malingering diagnosis leads to anxiety; Invisible disability | Quality of services<br>Beside manner | Environmental Factor | e5 Services, systems and policies | e580 Health services, systems and policies | e5809 Health services, systems and policies, other unspecified |
| *T02: "So I was following actually all of the recommendations and I wasn't getting better, no one considered my needs"* | TBI | Rehabilitation wasn't working; No progress | Individualized focus | Environmental Factor | e5 Services, systems and policies | e580 Health services, systems and policies | e5809 Health services, systems and policies, other unspecified |
| *C04: "we educate [clinicians working at acute setting] our patients with time management, because that has an impact into mobility, when to stop, when to start"* | Clinician | Providing education; strategies - time management that impact on mobility | Education services to patients<br>Quality of services | Environmental Factor | e5 Services, systems and policies | e580 Health services, systems and policies | e5808 Health services, systems and policies, other specified (education services) |
| *C010: "just doing a lot of teaching, the management, what is TBI and get a feel of how severe these symptoms are affecting them [at acute setting]"* | Clinician | Education as part of treatment planning | Education services to patients<br>Quality of services | Environmental Factor | e5 Services, systems and policies | e580 Health services, systems and policies | e5808 Health services, systems and policies, other specified (education services) |
| **1.2. Transition from acute to rehabilitation settings** | | | | | | | |
| *S03: "Everybody wants to get better. Everybody wants access* | Stroke | Access to services important, Motivated to get better | Accessibility to healthcare services | Environmental Factor | e5 Services, systems and policies | e580 Health services, systems and policies | e5809 Health services, systems and policies, other unspecified |

| Quotes | Population | Formulated statements | Codes | ICF domain | 1st Level ICF classification | 2nd Level ICF category | 3rd or higher Level ICF category |
|---|---|---|---|---|---|---|---|
| *to; we need access to health services"* | | | | | | | |
| *S01: "the occupational therapist at the xxx was very good and helpful"* | Stroke | Occupational therapy services help | Specialized services | Environmental Factor | e5 Services, systems and policies | e580 Health services, systems and policies | e5808 Health services, systems and policies, other specified (Specialized services) |
| *S03: "I found the physiotherapist very helpful and the occupational therapist over the speech therapy"* | Stroke | Physiotherapy and occupational therapy helpful and better than speech therapy | Specialized services | Environmental Factor | e5 Services, systems and policies | e580 Health services, systems and policies | e5808 Health services, systems and policies, other specified (Specialized services) |
| *S04: "For the next chunk of my experience, is the rehab places I went to xxx, everybody there was fantastic except for the speech therapist"* | Stroke | Some outpatient rehabilitation services is helpful | Accessibility to healthcare services Specialized services | Environmental Factor | e5 Services, systems and policies | e580 Health services, systems and policies | e5808 Health services, systems and policies, other specified (Specialized services) |
| *S04: "[the services] at xxx [rehabilitation centre] it was incredible, you talk about personalize service, it's incredible here"* | Stroke | Some outpatient rehabilitation services is helpful | Quality of services | Environmental Factor | e5 Services, systems and policies | e580 Health services, systems and policies | e5809 Health services, systems and policies, other unspecified |
| *S02:"the whole general system, it's the waiting to get to [rehabilitation], so you don't progress"* | Stroke | Wait times for services difficult; no progress while waiting | Accessibility to healthcare services Wait times | Environmental Factor | e5 Services, systems and policies | e580 Health services, systems and policies | e5809 Health services, systems and policies, other unspecified |
| *S02:"then you find yourself with a 2 month wait period and that's like okay"* | Stroke | Wait times for services difficult; no progress while waiting | Accessibility to healthcare services Wait times | Environmental Factor | e5 Services, systems and policies | e580 Health services, systems and policies | e5809 Health services, systems and policies, other unspecified |
| *S03: "as I probably speak mine, honestly unless you have like access to private"* | Stroke | Access to services/limited/ access to private | Accessibility to private healthcare services | Environmental Factor | e5 Services, systems and policies | e580 Health services, systems and policies | e5808 Health services, systems and policies, other specified (private services) |
| *S03: "reduce Wait times! I mean get people their needs and the proper treatments"* | Stroke | Wait times for services difficult; no progress while waiting | Accessibility to healthcare services Wait times | Environmental Factor | e5 Services, systems and policies | e580 Health services, systems and policies | e5809 Health services, systems and policies, other unspecified |
| *C06: "if the [patient] have a family support, to compensate, patients will be discharged faster, versus the person who is alone"* | Clinician | Discharge factors to community/family | Discharge factors Family support | Environmental Factor | e3 Support and relationships | e325 Acquaintances, peers, colleagues, neighbours and community members | |
| *C03:" we've got a huge waiting list"* | Clinician | Wait times impact on rehabilitation | Wait times | Environmental Factor | e5 Services, systems and policies | e580 Health services, systems and policies | e5809 Health services, systems and policies, other unspecified |
| *C04: "there is a gap between acute care and rehab"* | Clinician | Service Pathways from acute care to rehabilitation/limited | Service Pathways | Environmental Factor | e5 Services, systems and policies | e580 Health services, systems and policies | e5809 Health services, systems and policies, other unspecified |

| Quotes | Population | Formulated statements | Codes | ICF domain | 1st Level ICF classification | 2nd Level ICF category | 3rd or higher Level ICF category |
|---|---|---|---|---|---|---|---|
| *C03: "one of the challenges in outpatient rehab is we have to know when we stop treatment? And when they need to go to the next phase?"* | Clinician | Discharge guidelines from rehabilitation care to community Barriers: limited resources | Uncertainty | Not covered | | | |
| *C03:" the thing to bring in the table is in getting the discharge summaries, I mean that is not even knowledge transfer that's only information transfer"* | Clinician | Limits to discharge summaries; information vs. knowledge/Service Pathways from acute care to rehabilitation/limited | Service Pathways Workflow design | Environmental Factor | e5 Services, systems and policies | e580 Health services, systems and policies | e5808 Health services, systems and policies, other specified (workflow design) |
| *C01: "there is also another lack in the system is you know that the client either who never referred at all or the client who ends been through the continuum and never been discharge and there is no link"* | Clinician | Service Pathways from acute care to rehabilitation/limited | Service Pathways Workflow design | Environmental Factor | e5 Services, systems and policies | e580 Health services, systems and policies | e5808 Health services, systems and policies, other specified (workflow design) |
| *C03: "sometimes it takes a month to get the discharge summaries from outpatient to outpatient here even though it is written it is because they are Shortens archives"* | Clinician | Integration and infrastructure; limited resources; takes too long for information to move through system/Service Pathways from acute care to rehabilitation/limited | Service Pathways Workflow design | Environmental Factor | e5 Services, systems and policies | e580 Health services, systems and policies | e5808 Health services, systems and policies, other specified (workflow design) |
| *C06"sometimes the referral gave us the wrong information by saying everything is good about the patient with moderate or severe TBI, and a month later everything is going badly or it's really difficult for them to do the same tasks"* | Clinician | Barriers- some referral give the wrong information/Service Pathways from acute care to rehabilitation/limited | Service Pathways Workflow design | Environmental Factor | e5 Services, systems and policies | e580 Health services, systems and policies | e5808 Health services, systems and policies, other specified (workflow design) |
| *C04:" the young clients are mostly lost in the system and get no service [when] they are the ones who they have the most potential to go back to work"* | Clinician | Service Pathways from acute care to rehabilitation/limited to young clients | Service Pathways Young patients with impairments | Environmental Factor | e5 Services, systems and policies | e580 Health services, systems and policies | e5809 Health services, systems and policies, other unspecified |
| *C03: "talking about having you know informatics electronic health records, so if it there it is saves time"* | Clinician | Using electronic health records; saves time - mitigates limited resources | Electronic health records | Environmental Factor | e1 Products and technology | e198 Products and technology, other specified (EHRs) | |

| Quotes | Population | Formulated statements | Codes | ICF domain | 1st Level ICF classification | 2nd Level ICF category | 3rd or higher Level ICF category |
|---|---|---|---|---|---|---|---|
| *C03: "so they have not been able to put together the file and sending them by fax, but if it was electronic, [it] would be quick"* | Clinician | Electronic health records would improve flow and speed of information | Electronic health records | Environmental Factor | e1 Products and technology | e198 Products and technology, other specified (EHRs) | |
| *C01: "if we would be able to cut down on the paper work, we will have a whole of time transferring the information"* | Clinician | Better workflow; minimize paperwork | Electronic health records | Environmental Factor | e1 Products and technology | e198 Products and technology, other specified (EHRs) | |
| *C03: "but when people in a rehab bed if they only have mobility issue or if they have mobility issues and other things they are get into rehab"* | Clinician | Systemic challenges in service pathway from acute care to rehabilitation/ what deficit is priority | Service Pathways Deficits Discharge Factors | Environmental Factor not covered Activity and Participation | e5 Services, systems and policies<br><br>d4 Mobility | e580 Health services, systems and policies<br><br>d499 Mobility, unspecified | e5809 Health services, systems and policies, other unspecified |
| *C03:"but when they have visual or cognitive it can be pretty settle, they go home and they are not necessarily picked up until they have a serious physical problem"* | Clinician | Systemic challenges in service pathway from acute care to rehabilitation/ what deficit is priority | Service Pathways Deficits Discharge Factors | Environmental Factor Not covered Body Function | e5 Services, systems and policies b2 Sensory functions and pain b1 Mental Functions | e580 Health services, systems and policies b210 Seeing functions b144 Memory functions | e5809 Health services, systems and policies, other unspecified b2101 Visual field functions b1449 Memory functions, unspecified |
| *C04:"is the OT and inpatient going to evaluate if the person can read, no, can they see, can they navigate around or they are safe"* | Clinician | Systemic challenges in service pathway from acute care to rehabilitation/ what deficit is priority | Service Pathways Deficits Discharge Factors | Environmental Factor Not covered Activity and Participation | e5 Services, systems and policies d1 Learning and applying knowledge | e580 Health services, systems and policies Applying knowledge (d160-d179) | e5809 Health services, systems and policies, other unspecified |
| *C03: "when they go to inpatient how long they do need to stay inpatient before they discharged home"* | Clinician | Systemic challenges in service pathway from acute care to rehabilitation/uncertainty | Service Pathways Uncertainty Discharge Factors | Environmental Factor Not covered | e5 Services, systems and policies | e580 Health services, systems and policies | e5809 Health services, systems and policies, other unspecified |
| *C03: "the indicators of getting people to discharge as soon as somebody is able to be mobile and is able get to the bathroom and can do their ADLs they go home because they cannot keep staying there because otherwise they are going to block a bed and has repercussions"* | Clinician | Service Pathways from acute care to rehabilitation/Discharge Factors | Service Pathways Deficits Discharge Factors | Environmental Factor Not covered Activity and Participation | e5 Services, systems and policies d5 Self-Care | e580 Health services, systems and policies d599 Self-care, unspecified | e5809 Health services, systems and policies, other unspecified |
| *C02: "physically you say you need exercise to get better but cognitively well time well does the job, even for vision time will do the job no exercise, nothing"* | Clinician | Service Pathways from acute care to rehabilitation/Discharge Factors | Service Pathways Deficits Discharge Factors | Environmental Factor Not covered Activity and Participation Body Function | e5 Services, systems and policies d4 Mobility b1 Mental Functions | e580 Health services, systems and policies d499 Mobility, unspecified b144 Memory functions | e5809 Health services, systems and policies, other unspecified b1449 Memory functions, unspecified |

| Quotes | Population | Formulated statements | Codes | ICF domain | 1st Level ICF classification | 2nd Level ICF category | 3rd or higher Level ICF category |
|---|---|---|---|---|---|---|---|
| *C04: "if the [clients] had vision issues, vestibular issues, cognitive issues, or speech issues but physically they are fine, they will discharge with no service"* | Clinician | Service Pathways from acute care to rehabilitation/Discharge Factors | Service Pathways<br>Deficits<br>Discharge Factors | Environmental Factor<br>Not covered | e5 Services, systems and policies<br>b2 Sensory functions and pain<br>b3 Voice and speech functions<br>b1 Mental Functions | e580 Health services, systems and policies<br>b210 Seeing Functions<br>b330 Fluency and rhythm of speech functions<br>b144 Memory functions | e5809 Health services, systems and policies, other unspecified<br>b2101 Visual field functions<br>b3309 Fluency and rhythm of speech functions, unspecified<br>b1449 Memory functions, unspecified |
| *C01:"so that we are going really back in to acute care centers and [are] they doing proper triage"* | Clinician | Service Pathways from acute care to rehabilitation/Discharge Factors | Service Pathways<br>Discharge Factors | Environmental Factor | e5 Services, systems and policies | e580 Health services, systems and policies | e5809 Health services, systems and policies, other unspecified |
| *C01: "the education has to go back to the acute care teams in terms of rehabilitation"* | Clinician | Service pathways from acute care to rehabilitation limitation; education can be one strategy to improve care at acute level of care | Service Pathways<br>Education services to providers | Environmental Factor | e5 Services, systems and policies | e580 Health services, systems and policies | e5808 Health services, systems and policies, other specified (education services) |
| **1.3. Access to rehabilitation in the community setting** | | | | | | | |
| *C05: "when they are discharged, they can't go to the outpatient department because they are not independent to move around"* | Clinician | Discharge Factors/Deficits | Deficits<br>Discharge Factors | Not covered | | | |
| *C05:"it's like a grey area where that person would have had the potential to develop independence but it's not enough to keep it"* | Clinician | Limits to restoring independent functioning in community; limits to independency | Deficits<br>Discharge Factors | Not covered | | | |
| *C07: "To have the same services so you know to continue the rehabilitation outside of community, we need to have special services that can help them to function well at home"* | Clinician | Transition to community-based rehabilitation; limited resources impact on long-term functioning, mobility | Specialized services<br>Accessibility to healthcare services<br>Resources | Environmental Factor | e5 Services, systems and policies | e580 Health services, systems and policies<br>e590 Labour and employment services, systems and policies | e5808 Health services, systems and policies, other specified (specialized services)<br>e 5908 Labour and employment services, systems and policies, other specified (resources) |
| *C06: "Even for the stroke clientele, some patients don't have access to the CPA program after discharge"* | Clinician | lack of services; difficult to plan | Specialized services<br>Accessibility to healthcare services | Environmental Factor | e5 Services, systems and policies | e580 Health services, systems and policies | e5808 Health services, systems and policies, other specified (specialized services) |
| *S03: "I watched CLSC come to their house [disabled friend] and they don't show up on time"* | Stroke | Quality community-based services are lacking; not showing up on time | Specialized services<br>Accessibility to healthcare services | Environmental Factor | e5 Services, systems and policies | e580 Health services, systems and policies | e5808 Health services, systems and policies, other specified (specialized services) |

| Quotes | Population | Formulated statements | Codes | ICF domain | 1st Level ICF classification | 2nd Level ICF category | 3rd or higher Level ICF category |
|---|---|---|---|---|---|---|---|
| *C05: "I find it's the difficult area when our patients are discharged, when we know that he would have the potential to become independent but it's as if the services don't exist in a certain way or at least not in an optimal way to continue that with him"* | Clinician | Community-based - lack of health care services; difficult to plan | Specialized services<br>Resources<br>Deficits<br>Discharge Factors | Environmental Factor<br><br>Not covered | e5 Services, systems and policies | e580 Health services, systems and policies<br>e590 Labour and employment services, systems and policies | e5808 Health services, systems and policies, other specified (specialized services)<br>e 5908 Labour and employment services, systems and policies, other specified (resources) |
| **1.4. Re-integration into the community** | | | | | | | |
| *C05: "we can do training [for people with cognitive impairments at the clinic] but they will have difficulty making the connections in their real environment"* | Clinician | Care pathway: community-based - lack of services; difficult to plan | Re-integration<br>Cognition | Environmental Factor<br><br>Body Function | e5 Services, systems and policies<br><br>b1 Mental Functions | e580 Health services, systems and policies<br><br>b144 Memory functions | e5809 Health services, systems and policies, other unspecified |
| *T01:"my first occupational therapist wasn't really like telling me how to integrate myself really into regular life, like they were kind of just giving me some activities to do while I was there. And then we'd have a little talk but I didn't like it I didn't connect with them very much"* | TBI | Rapport with occupational therapy was difficult; There was a lack of integration of therapy into real life | Re-integration<br>Specialized services<br>Individualized focus | Environmental Factor | e5 Services, systems and policies | e580 Health services, systems and policies | e5808 Health services, systems and policies, other specified (specialized services) |
| *T01:"the second one I had he really focuses on each person well and he really gave us a schedule and he was the one who was like, okay, you need to push yourself. And I know it's not going to feel good and it's scary and everything"* | TBI | Rapport with 2nd professional was good; Scheduled, provided guidance and support and honest hard truths about path of recovery | Re-integration<br>Specialized services<br>Individualized focus | Environmental Factor | e5 Services, systems and policies | e580 Health services, systems and policies | e5808 Health services, systems and policies, other specified (specialized services) |
| *T01:"He [OT] was pushed me to go further than I thought I could which was reassuring telling you is okay like to do. I wasn't feeling like I was scared that I was like, doing something shouldn't be doing"* | TBI | Occupational therapy services was good; Pushed hard, progressed further that thought possible | Re-integration<br>Specialized services<br>Individualized focus | Environmental Factor | e5 Services, systems and policies | e580 Health services, systems and policies | e5808 Health services, systems and policies, other specified (specialized services) |
| *C06: "we try to put [the patients who are alone] in places [that offer] services to compensate for their safety"* | Clinician | Discharge planning; safety as priority; determining factors include support system (living alone) | Re-integration<br>Support services<br>Safety | Environmental Factor | e5 Services, systems and policies<br>e5 Services, systems and policies | e580 Health services, systems and policies<br>e530 Utilities services, systems and policies | e5808 Health services, systems and policies, other specified (support services)<br>e5308 Utilities services, systems and policies, other specified (Safety |

| Quotes | Population | Formulated statements | Codes | ICF domain | 1st Level ICF classification | 2nd Level ICF category | 3rd or higher Level ICF category |
|---|---|---|---|---|---|---|---|
| | | | | | | | standards for individuals with ABI) |
| *C06: "the person can be functional at home, but obviously [it is difficult to them] to be functional when they are going outdoors"* | Clinician | Discharge planning; safety as priority; determining factors include support system (living alone) | Re-integration<br>Support services<br>Safety | Environmental Factor | e5 Services, systems and policies<br>e5 Services, systems and policies | e580 Health services, systems and policies<br>e530 Utilities services, systems and policies | e5808 Health services, systems and policies, other specified (support services)<br>e5308 Utilities services, systems and policies, other specified (Safety standards for individuals with ABI) |
| *C05: "it is important to leave the patient with all the needed services when they are in community"* | Clinician | Transition to community-based rehab; limited resources impact on long-term functioning, mobility | Re-integration<br>Support services<br>Safety | Environmental Factor | e5 Services, systems and policies<br>e5 Services, systems and policies | e580 Health services, systems and policies<br>e530 Utilities services, systems and policies | e5808 Health services, systems and policies, other specified (support services)<br>e5308 Utilities services, systems and policies, other specified (Safety standards for individuals with ABI) |
| *S04:"Like I remember one of the classes, they are talking occupation, like if you got to fold clothes, don't stand over the table and do it, sit down and do it"* | Stroke | Structuring activities to be simple and take less energy facilitates participation and mobility | Re-integration<br>Education services to patients | Environmental Factor | e5 Services, systems and policies<br>e5 Services, systems and policies | e580 Health services, systems and policies<br>e530 Utilities services, systems and policies | e5809 Health services, systems and policies, other unspecified<br>e5308 Utilities services, systems and policies, other specified (Education services) |
| *S04: "the lady said well for your cooking, move your dishes that you used to this shower, if you tell me there's a normal person, I'd go what's the point, but you don't have the small battery"* | Stroke | Structuring activities to be simple facilitates participation and mobility | Re-integration<br>Education services to patients | Environmental Factor | e5 Services, systems and policies<br>e5 Services, systems and policies | e580 Health services, systems and policies<br>e530 Utilities services, systems and policies | e5809 Health services, systems and policies, other unspecified<br>e5308 Utilities services, systems and policies, other specified (Education services) |
| *T02:"there was a six week program and meditation and part of xxx education that I found very, very, very helpful. I really found it made the biggest shift in my education part"* | TBI | Meditation program was helpful | Re-integration<br>Education services to patients | Environmental Factor | e5 Services, systems and policies<br>e5 Services, systems and policies | e580 Health services, systems and policies<br>e530 Utilities services, systems and policies | e5809 Health services, systems and policies, other unspecified<br>e5308 Utilities services, systems and policies, other specified (Education services) |
| *C06: "[for persons who lives alone], it is important to make* | Clinician | Discharge planning; safety as priority; | Support services<br>Safety | Environmental Factor | e5 Services, systems and policies | e580 Health services, systems and policies | e5808 Health services, systems and policies, |

| Quotes | Population | Formulated statements | Codes | ICF domain | 1st Level ICF classification | 2nd Level ICF category | 3rd or higher Level ICF category |
|---|---|---|---|---|---|---|---|
| *sure that they are safe, so it's always a must to have the notion of safety at home"* | | determining factors include support system (living alone) | | | e5 Services, systems and policies | e530 Utilities services, systems and policies | other specified (support services) e5308 Utilities services, systems and policies, other specified (Safety standards for individuals with ABI) |
| **1.5. Follow-up in the community** | | | | | | | |
| *C03: "if they got a survey monkey of something to say are there any problems, how are you doing? Would help"* | Clinician | Follow up patients in the community using technology | Survey<br>Follow-up | Environmental Factor | e1 Products and technology | e198 Products and technology, other specified (survey) | |
| *C07: "We're not the team that can do the training at their environment, [and the service] may take six months, we don't have the recourses and service to train our patients at their home. I think that's one of our big problems"* | Clinician | Community-based - lack of services; difficult to plan | Support services<br>Recourses<br>Follow-up | Environmental Factor | e5 Services, systems and policies | e580 Health services, systems and policies<br>e590 Labour and employment services, systems and policies | e5808 Health services, systems and policies, other specified (support services)<br>e 5908 Labour and employment services, systems and policies, other specified (resources) |
| *C06: "in rehabilitation phase 2, the patient can go home once he is safe, and able to meet basic needs"* | Clinician | Eludes to some discharge planning standards (safety and basic needs) | Support services<br>Safety | Environmental Factor | e5 Services, systems and policies<br>e5 Services, systems and policies | e580 Health services, systems and policies<br>e530 Utilities services, systems and policies | e5808 Health services, systems and policies, other specified (support services)<br>e5308 Utilities services, systems and policies, other specified (Safety standards for individuals with ABI) |
| *C05: "we do follow-ups, which last between 6 months to sometimes 2 years"* | Clinician | Follow up patients in the community using phone | Follow-up | Environmental Factor | e5 Services, systems and policies | e580 Health services, systems and policies | e5809 Health services, systems and policies, other unspecified |
| *C06: "We do [the follow-up] more often by phone call, that would be useful for a certain category of people"* | Clinician | Follow up patients in the community using phone | Follow-up | Environmental Factor | e5 Services, systems and policies | e580 Health services, systems and policies | e5809 Health services, systems and policies, other unspecified |
| *C05: "we do the follow up for our clients"* | Clinician | Follow up: varies | Follow-up | Environmental Factor | e5 Services, systems and policies | e580 Health services, systems and policies | e5809 Health services, systems and policies, other unspecified |

| Quotes | Population | Formulated statements | Codes | ICF domain | 1st Level ICF classification | 2nd Level ICF category | 3rd or higher Level ICF category |
|---|---|---|---|---|---|---|---|
| *C05: "sometimes we do the follow up for other clients who came directly from the community and they are mobile"* | Clinician | Follow up: varies - source of referral | Follow-up | Environmental Factor | e5 Services, systems and policies | e580 Health services, systems and policies | e5809 Health services, systems and policies, other unspecified |
| *C05: "sometimes, we see improvements in our patients after a year and a half of follow up. Most of the time it is physically not cognitively"* | Clinician | Follow up: timing and duration varies; as does improvement with patients | Follow-up<br>Deficits | Environmental Factor<br>Not covered | e5 Services, systems and policies | e580 Health services, systems and policies | e5809 Health services, systems and policies, other unspecified |
| *C05: "It's not enough to provide, sometimes a guardianship, but that's it, it's just to have a little guidance that could help them, just to guide them"* | Clinician | Cognitive deficits may require guardianship; or supervision; or assistance | Support services<br>Safety<br>Deficits | Environmental Factor<br><br>not covered | e5 Services, systems and policies<br>e5 Services, systems and policies | e580 Health services, systems and policies<br>e530 Utilities services, systems and policies | e5808 Health services, systems and policies, other specified (support services)<br>e5308 Utilities services, systems and policies, other specified (Safety standards for individuals with ABI) |
| *C05: "to have immediate and intensive follow-up [to our patients who are discharged] and make transition between outpatient and home very smooth and accomplish patients needs at his own environment"* | Clinician | Transition btw stages of rehab; acute to community; what resources available | Follow-up | Environmental Factor | e5 Services, systems and policies | e580 Health services, systems and policies | e5809 Health services, systems and policies, other unspecified |
| *C06: "to take care of our patients in the long term especially for people who don't have the capacity to go back to work, how to keep them active?"* | Clinician | Long-term planning for activity/mobility; community-based treatment | Follow-up<br>Support services | Environmental Factor | e5 Services, systems and policies | e580 Health services, systems and policies | e5808 Health services, systems and policies, other specified (support services) |
| *C06: "to keep them as active as possible after discharge"* | Clinician | Long-term planning for activity/mobility; community-based treatment | Follow-up | Environmental Factor | e5 Services, systems and policies | e580 Health services, systems and policies | e5809 Health services, systems and policies, other unspecified |
| *C06: "the problem is who is going to do the follow up during the transition from phase 2 to 3"* | Clinician | Transition to community-based rehab; limited resources impact on long-term functioning, mobility | Follow-up | Environmental Factor | e5 Services, systems and policies | e580 Health services, systems and policies | e5809 Health services, systems and policies, other unspecified |
| *C06: "if we take charge to do the follow up and do the training immediately at their home after discharge, we will save time but* | Clinician | Transition to community-based rehab; limited resources impact | Follow-up<br>Support services<br>Resources | Environmental Factor | e5 Services, systems and policies | e580 Health services, systems and policies | e5808 Health services, systems and policies, other specified (support services) |

| Quotes | Population | Formulated statements | Codes | ICF domain | 1st Level ICF classification | 2nd Level ICF category | 3rd or higher Level ICF category |
|---|---|---|---|---|---|---|---|
| *the reality is that other clinician will take the responsibility and this will take a very long time"* | | on long-term functioning, mobility | | | | e590 Labour and employment services, systems and policies | e 5908 Labour and employment services, systems and policies, other specified (resources) |
| *C01: "offering tele-rehab to those clients during winter time"* | Clinician | Shifting services; secondary effect different mode of service delivery- adapting tele-rehabilitation to be able to maintain as much of in person as possible | Follow-up Tele-health | Environmental Factor | e5 Services, systems and policies e1 Products and technology | e580 Health services, systems and policies e198 Products and technology, other specified (tele-rehabilitation) | e5809 Health services, systems and policies, other unspecified |
| *C01: "it would be a limited session, a different type of session maybe but at least it is maintaining some contacts"* | Clinician | Shifting services; secondary effect different mode of service delivery- adapting tele-rehabilitation to be able to maintain as much of in person as possible | Follow-up Tele-health | Environmental Factor | e5 Services, systems and policies e1 Products and technology | e580 Health services, systems and policies e198 Products and technology, other specified (tele-rehabilitation) | e5809 Health services, systems and policies, other unspecified |
| *C05: "I always think that [tele-rehab is good] for patients that they just need supervision from time to time"* | Clinician | Tele-health; depends on deficit/task; Access to tele-health resources | Follow-up Tele-health | Environmental Factor | e5 Services, systems and policies e1 Products and technology | e580 Health services, systems and policies e198 Products and technology, other specified (tele-rehabilitation) | e5809 Health services, systems and policies, other unspecified |
| *C03: "Technology is more to follow up the patient to have data but the patient doesn't know"* | Clinician | Technology to help with transition to community-based care | Follow-up Technology | Environmental Factor | e5 Services, systems and policies e1 Products and technology | e580 Health services, systems and policies e199 Products and technology, other unspecified | e5809 Health services, systems and policies, other unspecified |
| *C03: "so if the patient has a smart watch that would help"* | Clinician | Wearable (smart watch) for rehab | Follow-up Technology | Environmental Factor | e5 Services, systems and policies e1 Products and technology | e580 Health services, systems and policies e198 Products and technology, other specified (smart watch) | e5809 Health services, systems and policies, other unspecified |

| Quotes | Population | Formulated statements | Codes | ICF domain | 1st Level ICF classification | 2nd Level ICF category | 3rd or higher Level ICF category |
|---|---|---|---|---|---|---|---|
| | | | | | | | |
| *C05: "it would be interesting to have, you know, a little chip that would allow us to see what route he took when he was really on his own"* | Clinician | Community-based assessment: use technology (RFI chip) | Follow-up Technology | Environmental Factor | e5 Services, systems and policies<br>e1 Products and technology | e580 Health services, systems and policies<br>e198 Products and technology, other specified (RFI chip) | e5809 Health services, systems and policies, other unspecified |
| *C02: "it could be virtual reality, kind of thing"* | Clinician | Virtual reality can be proper solution to treat patients during winter seasons | Follow-up Technology | Environmental Factor | e5 Services, systems and policies<br>e1 Products and technology | e580 Health services, systems and policies<br>e198 Products and technology, other specified (virtual reality) | e5809 Health services, systems and policies, other unspecified |
| *C06: "it's usually hard for older people to deal with a cell phone or a tablet or with a computer"* | Clinician | Age may impact usability, feasibility of using technology | Follow-up Technology | Environmental Factor | e5 Services, systems and policies<br>e1 Products and technology | e580 Health services, systems and policies<br>e199 Products and technology, other unspecified | e5809 Health services, systems and policies, other unspecified |
| *C06: "[using a tele-rehab] is not safe to cross the streets. Maybe it's to organize routines and things like that, in the house, but you know, to go outside for training, when you ask for supervision, it takes someone there, physically present"* | Clinician | Tele-health; depends on deficit/task; Limits to using tele-health - need to use in concert with in-person | Follow-up<br>Safety<br>Tele-health | Environmental Factor | e5 Services, systems and policies<br><br>e1 Products and technology | e580 Health services, systems and policies<br>e530 Utilities services, systems and policies<br>e198 Products and technology, other specified (tele-rehabilitation) | e5809 Health services, systems and policies, other unspecified<br>e5308 Utilities services, systems and policies, other specified (Safety standards for individuals with ABI) |
| *C05: "but for patients who are not completely safe, who are at risk of falling at home, tele-rehab would not work because I think it is only important to do the follow-up interventions"* | Clinician | Tele-health; depends on deficit/task; limits to telehealth; need for multi-modal treatment | Follow-up<br>Safety<br>Tele-health | Environmental Factor | e5 Services, systems and policies | e580 Health services, systems and policies<br>e530 Utilities services, systems and policies | e5809 Health services, systems and policies, other unspecified<br>e5308 Utilities services, systems and policies, other |

| Quotes | Population | Formulated statements | Codes | ICF domain | 1st Level ICF classification | 2nd Level ICF category | 3rd or higher Level ICF category |
|---|---|---|---|---|---|---|---|
| | | | | | e1 Products and technology | e198 Products and technology, other specified (tele-rehabilitation) | specified (Safety standards for individuals with ABI) |
| *C05: "I honestly don't think so that virtual reality would work because the generalization point of view you know to do that in the lab is not same as real life, it doesn't transpose in the same way"* | Clinician | Virtual reality - does it generalize to real life; Need to use multi-modal approach to treatment | Follow-up<br>Safety<br>Technology | Environmental Factor | e5 Services, systems and policies<br><br>e1 Products and technology | e580 Health services, systems and policies<br>e530 Utilities services, systems and policies<br><br>e198 Products and technology, other specified (virtual reality) | e5809 Health services, systems and policies, other unspecified<br>e5308 Utilities services, systems and policies, other specified (Safety standards for individuals with ABI) |
| *C06: "Perhaps it works with the clientele with little deficit, which is a good way to stimulate or to see, but I am certain that, as such, the clientele that is a little more cognitively affected, and the clientele that is a little more rigid, that would be a little harder"* | Clinician | Virtual reality - limits to applicability for all patients; Need to use multi-modal approach to treatment | Follow-up<br>Safety<br>Technology | Environmental Factor | e5 Services, systems and policies<br><br>e1 Products and technology | e580 Health services, systems and policies<br>e530 Utilities services, systems and policies<br><br>e198 Products and technology, other specified (virtual reality) | e5809 Health services, systems and policies, other unspecified<br>e5308 Utilities services, systems and policies, other specified (Safety standards for individuals with ABI) |
| *C05: "it's over 60% of our entire clientele, older people with mild TBI and we have hard time following them up"* | Clinician | Follow up: varies - by age | Follow-up | Environmental Factor | e5 Services, systems and policies | e580 Health services, systems and policies | e5809 Health services, systems and policies, other unspecified |
| *C06: "We don't usually follow up young adults with mild TBI who are either going back to work or who have a family"* | Clinician | Follow up: varies - by age and function | Follow-up | Environmental Factor | e5 Services, systems and policies | e580 Health services, systems and policies | e5809 Health services, systems and policies, other unspecified |

| Quotes | Population | Formulated statements | Codes | ICF domain | 1st Level ICF classification | 2nd Level ICF category | 3rd or higher Level ICF category |
|---|---|---|---|---|---|---|---|
| *C05: "30% with moderate to severe TBI may be better to have them here than they leave to their home"* | Clinician | Follow up: varies - by age and function | Follow-up | Environmental Factor | e5 Services, systems and policies | e580 Health services, systems and policies | e5809 Health services, systems and policies, other unspecified |
| *C06: "I still have a gentleman who has just had a leave of absence and we're a bit uncertain, even if this gentleman can pick up the phone and talk and remember exactly who I am and why, it's not as simple as that"* | Clinician | Follow-up; but serious deficits and other factors may not be overcome by technology | Follow-up<br>Safety<br>Deficits | Environmental Factor<br><br>not covered | e5 Services, systems and policies | e580 Health services, systems and policies<br><br>e530 Utilities services, systems and policies | e5809 Health services, systems and policies, other unspecified<br>e5308 Utilities services, systems and policies, other specified (Safety standards for individuals with ABI) |
| **Theme 2: System Design** | | | | | | | |
| **2.1. Quality of care** | | | | | | | |
| *T01: I felt like the doctors that I saw didn't really know, like they didn't have very good suggestions of what to do and how to help"* | TBI | Lack of quality doctor services | Knowledge acquisition<br>Quality of services | Environmental Factor | e5 Services, systems and policies | e580 Health services, systems and policies | e5809 Health services, systems and policies, other unspecified |
| *T01: "I think they [doctors] Kind of didn't really understand how it was really affecting [i.e. TBI] my life and didn't really kind of know how to guide me where to go"* | TBI | Lack of quality services; lack of knowledge of impact of traumatic brain injury; Lack of guidance | Knowledge acquisition<br>Quality of services | Environmental Factor | e5 Services, systems and policies | e580 Health services, systems and policies | e5809 Health services, systems and policies, other unspecified |
| *T01: "They kind of said go to physio, go to occupational therapy and like, that's kind of it, and it caused me a lot of anxiety and then he [the doctor] just was like, here's some anxiety meds!"* | TBI | Bedside manner poor; Just access services with lack of explanation and guidance; Medical model | Knowledge acquisition<br>Quality of services | Environmental Factor | e5 Services, systems and policies | e580 Health services, systems and policies | e5809 Health services, systems and policies, other unspecified |
| *T02: "family doctor didn't know much, he was giving me a medication that wasn't working."* | TBI | Lack of traumatic brain injury knowledge; quality of services; Medical Model | Knowledge acquisition<br>Quality of services | Environmental Factor | e5 Services, systems and policies | e580 Health services, systems and policies | e5809 Health services, systems and policies, other unspecified |
| *T01:"And I think like in terms of the doctor didn't really know how to handle that and like it's hard because like all these professionals like there. This is their job"* | TBI | Lack of traumatic brain injury knowledge, Quality of services | Knowledge acquisition<br>Quality of services | Environmental Factor | e5 Services, systems and policies | e580 Health services, systems and policies | e5809 Health services, systems and policies, other unspecified |
| *T01: "At the same time, if they haven't experienced it like it's also hard to help you"* | TBI | Lack of traumatic brain injury knowledge, Quality of services | Knowledge acquisition<br>Quality of services | Environmental Factor | e5 Services, systems and policies | e580 Health services, systems and policies | e5809 Health services, systems and policies, other unspecified |

| Quotes | Population | Formulated statements | Codes | ICF domain | 1st Level ICF classification | 2nd Level ICF category | 3rd or higher Level ICF category |
|---|---|---|---|---|---|---|---|
| *T03:"I guess I just wish in some way that when they sort of discovered that you have a mild concussion that they understand that it's still as debilitating as maybe what they would consider a more serious concussion"* | TBI | Uncertainty of impact of deficits; perception of a diagnosis; education of healthcare professionals | Knowledge acquisition<br>Quality of services | Environmental Factor | e5 Services, systems and policies | e580 Health services, systems and policies | e5809 Health services, systems and policies, other unspecified |
| *T01:"but I feel like nobody could really give you good education or like information about what was going on, they kind of just, you know, give you short answers that kind of thing"* | TBI | Guidelines for treatment; Education and information for patients; Staying up to date is difficult | Knowledge acquisition<br>Quality of services | Environmental Factor | e5 Services, systems and policies | e580 Health services, systems and policies | e5809 Health services, systems and policies, other unspecified |
| *T01:"they definitely help [the healthcare providers] with like symptom management so symptom management was obviously like one of the biggest things"* | TBI | Symptom management useful in rehabilitation | Symptom management<br>Quality of services | Environmental Factor | e5 Services, systems and policies | e580 Health services, systems and policies | e5808 Health services, systems and policies, other specified (symptom management) |
| *T01:"my Osteo and the Cairo are the ones that really like actually helped a lot with my symptoms"* | TBI | Osteopath, Chiropractor useful in rehabilitation | Symptom management<br>Quality of services | Environmental Factor | e5 Services, systems and policies | e580 Health services, systems and policies | e5808 Health services, systems and policies, other specified (symptom management) |
| *S03: "because she said there's only so much she can do for me and I had to do it on my own [the speech therapist], I was discouraged actually to hear when she said that to me, I don't know, I could have wanted more"* | Stroke | Bedside manner of speech therapy discouraging; I wanted to improve; Loss of hope | Specialized Therapy<br>Individualized focus<br>Quality of services | Environmental Factor | e5 Services, systems and policies | e580 Health services, systems and policies | e5808 Health services, systems and policies, other specified (specialized services) |
| *T03:"What are those steps and How to apply them, you really need a specialist to really get in there and focus and help you improve on very specific things and how to do those things"* | TBI | Specialized professionals; Individualized treatment would be useful | Specialized Therapy<br>Individualized focus<br>Quality of services | Environmental Factor | e5 Services, systems and policies | e580 Health services, systems and policies | e5808 Health services, systems and policies, other specified (specialized services) |
| *T02:"They told me I finished the rehab at xxx and I feel really left in the cold, I do not feel ready to be in the world at all, so what is next for me?"* | TBI | Lack of shared decision making | Specialized Therapy<br>Individualized focus<br>Quality of services | Environmental Factor | e5 Services, systems and policies | e580 Health services, systems and policies | e5808 Health services, systems and policies, other specified (specialized services) |
| *T02: "So we set some goals they never said after you finish those goals or you're going to be finished"* | TBI | Lack of education about transition to end of services | Individualized focus<br>Quality of services | Environmental Factor | e5 Services, systems and policies | e580 Health services, systems and policies | e5809 Health services, systems and policies, other unspecified |

| Quotes | Population | Formulated statements | Codes | ICF domain | 1st Level ICF classification | 2nd Level ICF category | 3rd or higher Level ICF category |
|---|---|---|---|---|---|---|---|
| *T04: "I was there [at rehabilitation centre] for the stroke not for the pain right, so they didn't take the pain into consideration at all"* | TBI | lack of individualized focus | Specialized Therapy Quality of services | Environmental Factor | e5 Services, systems and policies | e580 Health services, systems and policies | e5808 Health services, systems and policies, other specified (specialized services) |
| *T05: "so for me personally i think that xxx should not be a place for rehabilitation for those with a TBI"* | TBI | Some institutions are better for specific injuries and healthcare | Specialized Therapy | Environmental Factor | e5 Services, systems and policies | e580 Health services, systems and policies | e5808 Health services, systems and policies, other specified (specialized services) |
| *S03: "I was considered high functioning when I got here at xxx"* | Stroke | Impact of status and diagnosis on access to services | Specialized Therapy | Environmental Factor | e5 Services, systems and policies | e580 Health services, systems and policies | e5808 Health services, systems and policies, other specified (specialized services) |
| *C01: "I have some real concerns about [the health system] down the road even getting like worse with like the elderly you know, like it should have been more of a demand"* | Clinician | System of care not good enough for the patient; particularly the elderly | Healthcare services Quality of services | Environmental Factor | e5 Services, systems and policies | e580 Health services, systems and policies | e5809 Health services, systems and policies, other unspecified |
| *S04: "It's good though, I found that the rehab centers are good, the doctors well good luck with that one! And then, it's actually service that are missed still"* | Stroke | Services range from bad to good; Also, some services are missing | Healthcare services Quality of services | Environmental Factor | e5 Services, systems and policies | e580 Health services, systems and policies | e5809 Health services, systems and policies, other unspecified |
| *S03: "We have a very broken system. I have to tell you, none of the people sitting here don't not want to get better"* | Stroke | System of care not good enough for the patient | Healthcare services Quality of services | Environmental Factor | e5 Services, systems and policies | e580 Health services, systems and policies | e5809 Health services, systems and policies, other unspecified |
| *S03: "the system is very broken, it's very broken, and it's very sad, and it's very discouraging"* | Stroke | System of care not good enough for the patient | Healthcare services Quality of services | Environmental Factor | e5 Services, systems and policies | e580 Health services, systems and policies | e5809 Health services, systems and policies, other unspecified |
| **2.2. Information services** | | | | | | | |
| *T01: "I don't know, it's kind of hard to navigate what to do because you didn't really know who to listen to for where to turn"* | TBI | Navigate system; Lack of case management service; System | Navigate system Case management services Information services | Environmental Factor | e5 Services, systems and policies | e580 Health services, systems and policies | e5809 Health services, systems and policies, other unspecified |
| *T01: But so I really had to do a lot like on my own to try and find therapists and I went through everything you know like physio, occupational therapy, acupuncture, osteo, chiropractic"* | TBI | Navigate system; Lack of case management service; System | Navigate system Case management services Information services | Environmental Factor | e5 Services, systems and policies | e580 Health services, systems and policies | e5809 Health services, systems and policies, other unspecified |

| Quotes | Population | Formulated statements | Codes | ICF domain | 1st Level ICF classification | 2nd Level ICF category | 3rd or higher Level ICF category |
|---|---|---|---|---|---|---|---|
| *T01:"“I did everything, you know, so I had to really find all these people on my own, and so it was hard to navigate, it takes time”* | TBI | Navigate system; Lack of case management service; System | Navigate system<br>Case management services<br>Information services | Environmental Factor | e5 Services, systems and policies | e580 Health services, systems and policies | e5809 Health services, systems and policies, other unspecified |
| *S04: “Accountability and oversight! who is he going to complain to? The manager?”* | Stroke | Oversight and accountability needed | Case management services<br>Accountability | Environmental Factor | e5 Services, systems and policies<br>e1 Products and Technology | e580 Health services, systems and policies<br>e165 Assets | e5809 Health services, systems and policies, other unspecified<br>e198 Products and technology, other specified (accountability) |
| *S04: “there is no bigger oversight. First one was a stroke, here is neurologist, here is a test, here is this place, the para-transit, there is nobody taking care of it all”* | Stroke | Oversight and accountability needed; Case management services would be useful | Case management services<br>Accountability<br>Information services | Environmental Factor | e5 Services, systems and policies<br>e1 Products and Technology | e580 Health services, systems and policies<br>e165 Assets | e5809 Health services, systems and policies, other unspecified<br>e198 Products and technology, other specified (accountability) |
| *S01: “where do I get that information or How do I get, you know this or like, anticipatory guidance”* | Stroke | Lack of data infrastructure; Processes are lacking | Information services | Environmental Factor | e5 Services, systems and policies | e580 Health services, systems and policies | e5809 Health services, systems and policies, other unspecified |
| *T03: "my concussion was considered mild, but my therapist at xxx said sometimes there's a small percentage of cases with mild that just go on a very long time" "And that's why I've been sent there for them to work on some long term solutions"* | TBI | Mild may not be so mild as it may last a long time; Service provided difficult but useful information | Information services | Environmental Factor | e5 Services, systems and policies | e580 Health services, systems and policies | e5809 Health services, systems and policies, other unspecified |
| *T03:"I joined a group at xxx that was like it was in the Information Group about how concussions are different from traumatic brain injuries"* | TBI | Support group/ information group useful | Information services | Environmental Factor | e5 Services, systems and policies | e580 Health services, systems and policies | e5809 Health services, systems and policies, other unspecified |
| **2.3. Oriented teamwork approach** | | | | | | | |
| *S03: “Some are really stressed out, in the health care system , they may start off with more ideal vision of what they like, and then in the end they end up being you know, overworked”* | Stroke | Overworked healthcare professionals make it difficult | Overworked Professionals<br>Quality of services | Environmental Factor | e5 Services, systems and policies | e580 Health services, systems and policies | e5809 Health services, systems and policies, other unspecified |
| *S02: “Forced overtime, and too many patients on one nurse and the nurses doing the doctors job”* | Stroke | Overworked healthcare professionals make it difficult | Overworked Professionals<br>Quality of services | Environmental Factor | e5 Services, systems and policies | e580 Health services, systems and policies | e5809 Health services, systems and policies, other unspecified |

| Quotes | Population | Formulated statements | Codes | ICF domain | 1st Level ICF classification | 2nd Level ICF category | 3rd or higher Level ICF category |
|---|---|---|---|---|---|---|---|
| *S04: "the Healthcare in Canada is great, but the disconnect with the doctor and the others is the problem"* | Stroke | Disconnect between doctors and other professionals makes it difficult | Team-Oriented Quality of services | Environmental Factor | e5 Services, systems and policies | e580 Health services, systems and policies | e5809 Health services, systems and policies, other unspecified |
| *S04: "And you have the doctors, everybody is broken up the little parts and they don't talk to each other really"* | Stroke | Lack of quality team service | Team-Oriented Quality of services | Environmental Factor | e5 Services, systems and policies | e580 Health services, systems and policies | e5809 Health services, systems and policies, other unspecified |
| *T01: "when I do more therapy like everyone kind of has their own suggestions and each different field has their own suggestions"* | TBI | Lack of team oriented approach | Team-Oriented Quality of services | Environmental Factor | e5 Services, systems and policies | e580 Health services, systems and policies | e5809 Health services, systems and policies, other unspecified |
| *T01:"I mean, it depended like some of them [i.e. healthcare provider] said like you should do more therapy"; "some of them said you should do less like the doctors just kind of, I don't know"* | TBI | Lack of team-oriented approach | Team-Oriented Quality of services | Environmental Factor | e5 Services, systems and policies | e580 Health services, systems and policies | e5809 Health services, systems and policies, other unspecified |
| *T04: "everyone [healthcare providers] just kind of give their own opinions"* | TBI | Lack of team-oriented approach | Team-Oriented Quality of services | Environmental Factor | e5 Services, systems and policies | e580 Health services, systems and policies | e5809 Health services, systems and policies, other unspecified |
| *T01: "I didn't really feel that anybody really has a good guide of what you should really do"* | TBI | Lack of team-oriented approach | Team-Oriented Quality of services | Environmental Factor | e5 Services, systems and policies | e580 Health services, systems and policies | e5809 Health services, systems and policies, other unspecified |
| *T03: "It was just not super contradicting but difference of opinions and you kind of don't really know exactly what to listen to"* | TBI | Lack of team-oriented approach | Team-Oriented Quality of services | Environmental Factor | e5 Services, systems and policies | e580 Health services, systems and policies | e5809 Health services, systems and policies, other unspecified |
| *T02: "I was told I had a concussion. and, shouldn't do any exercise for two weeks, which I did"* | TBI | Guidelines for treatment inconsistent; no exercise for 2 weeks | Team-Oriented Quality of services | Environmental Factor | e5 Services, systems and policies | e580 Health services, systems and policies | e5809 Health services, systems and policies, other unspecified |
| *T02: "And then I was told to stay in a dark room for five days which I did"* | TBI | Guidelines for treatment inconsistent; stay in dark for 5 days | Team-Oriented Quality of services | Environmental Factor | e5 Services, systems and policies | e580 Health services, systems and policies | e5809 Health services, systems and policies, other unspecified |
| *T02: "And then I saw my family doctor and he says, Okay, you should be off of work for a month in which I did"* | TBI | Guidelines for treatment inconsistent; no work for a month | Team-Oriented Quality of services | Environmental Factor | e5 Services, systems and policies | e580 Health services, systems and policies | e5809 Health services, systems and policies, other unspecified |
| *S06: "Proper training for the provider and teach them how to take care of the old man"* | Stroke | Quality healthcare; education needed for providers | Education to providers Quality of services | Environmental Factor | e5 Services, systems and policies | e580 Health services, systems and policies | e5809 Health services, systems and policies, other unspecified |
| *T01: "I think maybe like education [to the healthcare providers] about what exactly is* | TBI | Guidelines for treatment; Education and information for patients; | Education to providers Quality of services | Environmental Factor | e5 Services, systems and policies | e580 Health services, systems and policies | e5809 Health services, systems and policies, other unspecified |

| Quotes | Population | Formulated statements | Codes | ICF domain | 1st Level ICF classification | 2nd Level ICF category | 3rd or higher Level ICF category |
|---|---|---|---|---|---|---|---|
| *going on and how they like see them like evolution like recovery"* | | Staying up to date is difficult | | | | | |
| *T01:"Maybe this is kind of what we think will happen, or how we can think get better, or how to Personalized the different kinds of therapies to each situation instead of just saying go try this"* | TBI | Guidelines for treatment; Education and information for patients; Staying up to date is difficult | Education to providers Quality of services | Environmental Factor | e5 Services, systems and policies | e580 Health services, systems and policies | e5809 Health services, systems and policies, other unspecified |
| *T01: "there's so many different information and everything changes a lot. The view of how to treat concussions and how they really like impact you have changed a lot over the last few years"* | TBI | Guidelines for treatment; Education and information for patients; Staying up to date is difficult | Education to providers Team-Oriented Quality of services | Environmental Factor | e5 Services, systems and policies | e580 Health services, systems and policies | e5809 Health services, systems and policies, other unspecified |
| **2.4. Self-management** | | | | | | | |
| *T01:"Maybe just like self-management at home with the link residual effects"* | TBI | Self-education about TBI; self-management of symptoms | Self-therapy | Environmental Factor | e5 Services, systems and policies | e580 Health services, systems and policies | e5809 Health services, systems and policies, other unspecified |
| *S03: "[the work] fell on me as well to make sure that I continue everything that I learned, you know and practicing it";"and I really push through that to bridge my gap"* | Stroke | Therapy left to patient to maintain, makes it difficult | Self-therapy | Environmental Factor | e5 Services, systems and policies | e580 Health services, systems and policies | e5809 Health services, systems and policies, other unspecified |
| *S03: "it's hard because if it falls so much on yourself, you're looking for help"* | Stroke | Therapy left to patient to maintain, makes it difficult | Self-therapy | Environmental Factor | e5 Services, systems and policies | e580 Health services, systems and policies | e5809 Health services, systems and policies, other unspecified |
| *S02: "I was lucky I had this therapist [speech therapist], I would have homework"* | Stroke | Speech therapy services provide homework and it's helpful | Self-therapy | Environmental Factor | e5 Services, systems and policies | e580 Health services, systems and policies | e5809 Health services, systems and policies, other unspecified |
| *S03: "I was just happy to get the help and to be told what to do a little bit you know, to know what to practice, to know how to stand, or walk"* | Stroke | Grateful for services and self-therapy | Self-therapy | Environmental Factor | e5 Services, systems and policies | e580 Health services, systems and policies | e5809 Health services, systems and policies, other unspecified |
| *T03:"And I've done a lot of research on my own, but how to deal with the day to day life stuff. That perhaps you can improve your symptoms during this day to day stuff"* | TBI | Self-education about TBI; Improve symptoms and their impact on ADLs | Self-therapy | Environmental Factor | e5 Services, systems and policies | e580 Health services, systems and policies | e5809 Health services, systems and policies, other unspecified |
| *T02: "[self-management and writing] helped me to work with my symptoms like you know pacing, managing symptoms, and it also helps with the scheduling"* | TBI | Self-management; writing, pacing, scheduling all helped | Self-therapy | Environmental Factor | e5 Services, systems and policies | e580 Health services, systems and policies | e5809 Health services, systems and policies, other unspecified |

| Quotes | Population | Formulated statements | Codes | ICF domain | 1st Level ICF classification | 2nd Level ICF category | 3rd or higher Level ICF category |
|---|---|---|---|---|---|---|---|
| *T02: "And my daily activities, whether it was my internet, shopping center, going into anywhere that reveals noise and very sensitive to all of the music fleeing blast in every single store every single shopping center I go around with headphones now"* | TBI | Noise-cancelling headphones are helpful in these environments | Self-therapy | Environmental Factor | e5 Services, systems and policies | e580 Health services, systems and policies | e5809 Health services, systems and policies, other unspecified |
| **Theme 3. Accessibility and services in the community** | | | | | | | |
| *S04: "So, you have the street, cement barrier, and the bicycle path and then the sidewalks, so, I am very curious, how does a handicap transfer"* | Stroke | Environmental barriers in the city limit mobility | Accessibility design Policy | Environmental Factor | e1 Products and technology<br><br>e5 Services, systems and policies | e150 Design, construction and building products and technology of buildings for public use<br>e515 Architecture and construction services, systems and policies | e 1500 Design, construction and building products and technology for entering and exiting buildings for public use<br>e 5152 Architecture and construction policies |
| *S04: "I find the city of Montreal, during the snowstorm, they don't care if you are a handicap person, that's it, you are stuck inside"* | Stroke | Weather (snow) and lack of services to keep pathways clean and accessible impact mobility | Accessibility design Policy | Environmental Factor | e1 Products and technology<br><br>e5 Services, systems and policies | e150 Design, construction and building products and technology of buildings for public use<br>e515 Architecture and construction services, systems and policies | e 1500 Design, construction and building products and technology for entering and exiting buildings for public use<br>e 5152 Architecture and construction policies |
| *S01:"[in the metro station] there is no escalators right, so it's like stairs, so how are you supposed to get down there, if like we do have a walker, you know. So, there's a limitation"* | Stroke | Lack of escalators in metro; impact on mobility | Accessibility design Policy | Environmental Factor | e1 Products and technology<br><br>e5 Services, systems and policies | e150 Design, construction and building products and technology of buildings for public use<br>e515 Architecture and construction services, systems and policies | e 1500 Design, construction and building products and technology for entering and exiting buildings for public use<br>e 5152 Architecture and construction policies |
| *S04: "I think the laws about handicap service is disabled"* | Stroke | Challenges with knowing the law; how to access services with appropriate diagnoses | Policy | Environmental Factors | e5 Services, systems and policies | e575 General social support services, systems and policies<br><br>e515 Architecture and construction services, systems and policies | e 5758 General social support services, systems and policies, other specified (handicap services)<br><br>e 5152 Architecture and construction policies |

| Quotes | Population | Formulated statements | Codes | ICF domain | 1st Level ICF classification | 2nd Level ICF category | 3rd or higher Level ICF category |
|---|---|---|---|---|---|---|---|
| *C03: "construction is a big thing and navigating, especially if you have clients have cognition issue on top of that, they have hard time figuring out how to do it"* | Clinician | Barriers; construction sites (uneven ground, safety issues); limited access due to environment | Accessibility design<br>Policy | Environmental Factor | e1 Products and technology<br><br>e5 Services, systems and policies | e150 Design, construction and building products and technology of buildings for public use<br>e515 Architecture and construction services, systems and policies | e 1500 Design, construction and building products and technology for entering and exiting buildings for public use<br>e 5152 Architecture and construction policies |
| *C04: "the street is clear but the sidewalks were not [because of the snow], so that's make it hard for the clients"* | Clinician | Community barriers cause a shift in mobility (wailing aid to wheelchair)y | Accessibility design<br>Policy | Environmental Factor | e1 Products and technology<br><br>e5 Services, systems and policies | e150 Design, construction and building products and technology of buildings for public use<br>e515 Architecture and construction services, systems and policies | e 1500 Design, construction and building products and technology for entering and exiting buildings for public use<br>e 5152 Architecture and construction policies |
| *S05: "I have bars in my house, so I don't use a cane inside; I got bars in the shower, now I don't have to use a bench anymore"* | Stroke | Accessible design features in the home (bars) help with mobility within the home | Accessibility design<br>Policy | Environmental Factor | e1 Products and technology<br><br>e5 Services, systems and policies | e150 Design, construction and building products and technology of buildings for public use<br>e515 Architecture and construction services, systems and policies | e 1500 Design, construction and building products and technology for entering and exiting buildings for public use<br>e 5152 Architecture and construction policies |
| *S05: "they would not let me do it [the shower] without a bench. And, they would not let me home [the hospital] until I promise, that I would put a bench"* | Stroke | Accessible design features in the home (shower) help with mobility within the home | Accessibility design<br>Policy | Environmental Factor | e1 Products and technology<br><br>e5 Services, systems and policies | e150 Design, construction and building products and technology of buildings for public use<br>e515 Architecture and construction services, systems and policies | e 1500 Design, construction and building products and technology for entering and exiting buildings for public use<br>e 5152 Architecture and construction policies |
| *T02"Well they close the gym [because of COVID]. So I had just started, I started just walking on the treadmill. I was a runner before so I can run"* | TBI | Disruptions in community services makes it hard to maintain progress | Recreational services | Environmental Factors | e5 Services, systems and policies | e575 General social support services, systems and policies | e 5758 General social support services, systems and policies, other specified (recreation services) |
| *T02: "it will be difficult [to acess to community services] because they were asking [the healthcare* | TBI | Difficult to work on rehab goals on your own | Accessibility design<br>Policy | Environmental Factor | e1 Products and technology | e150 Design, construction and building products and | e 1500 Design, construction and building products and |

| Quotes | Population | Formulated statements | Codes | ICF domain | 1st Level ICF classification | 2nd Level ICF category | 3rd or higher Level ICF category |
|---|---|---|---|---|---|---|---|
| *providers] you to do biking, hiking as a means to build up my cardio little bit"* | | | | | e5 Services, systems and policies | technology of buildings for public use<br>e515 Architecture and construction services, systems and policies | technology for entering and exiting buildings for public use<br>e 5152 Architecture and construction policies |
| *C02:" I think that the intervention doesn't need to be with clients in itself, but with the city and the community"* | Clinician | Policy strategies at the community level; systems thinking lens transport (parking) & access to places (malls…); built environment; policy | Accessibility design Policy | Environmental Factor | e1 Products and technology<br><br>e5 Services, systems and policies | e150 Design, construction and building products and technology of buildings for public use<br>e515 Architecture and construction services, systems and policies | e 1500 Design, construction and building products and technology for entering and exiting buildings for public use<br>e 5152 Architecture and construction policies |
| *C02:"can the city of Montreal know a bit more the places where is a parking lot for a handicap person can be a priority?"* | Clinician | Policy strategies at the community level; systems thinking lens transport (parking) & access to places (malls…); built environment; policy | Accessibility design Policy | Environmental Factor | e1 Products and technology<br><br>e5 Services, systems and policies | e150 Design, construction and building products and technology of buildings for public use<br>e515 Architecture and construction services, systems and policies | e 1500 Design, construction and building products and technology for entering and exiting buildings for public use<br>e 5152 Architecture and construction policies |
| **Theme 4: Transportation services** | | | | | | | |
| *S05:"I couldn't put a seat belt, it was impossible, you couldn't put it up, nobody in the back seat could put it up, [while using transport adapted]"* | Stroke | Transport adapted/ poor service/ there is no differentiation between mental and physical handicaps/long ride/many people at the back seat/Physical limitations, not being able to put on a seatbelt; lack of services to help | Transport adapted services | Environmental Factors | e5 Services, systems and policies | e540 Transportation services, systems and policies | e 5408 Transportation services, systems and policies, other specified (transport adapted) |
| *S05:"At first I couldn't handle buses or metro, I'd get overwhelmed with all the simulation and be sick"* | Stroke | Sensitivity to stimulation, information, noise, light impact on mobility | Public transportation services | Environmental Factor | e5 Services, systems and policies | e540 Transportation services, systems and policies | e 5408 Transportation services, systems and policies, other specified (Public transportation) |
| *S05:"I can do the buses for a decent amount now, as long as it's not in rush hour or something"* | Stroke | Tolerance to crowds, with community transit can be a barrier | Public transportation services | Environmental Factor | e5 Services, systems and policies | e540 Transportation services, systems and policies | e 5408 Transportation services, systems and policies, other specified (Public transportation) |

| Quotes | Population | Formulated statements | Codes | ICF domain | 1st Level ICF classification | 2nd Level ICF category | 3rd or higher Level ICF category |
|---|---|---|---|---|---|---|---|
| *S05: "and I know they, just to come here for an appointment of 11:00 o'clock, I should be ready at 8:15, they drive you around for 2 hours, like crammed in the back of a hatchback with two other people, [while using transport adapted]"* | Stroke | Challenges with adapting to using services; requires pre-planning, spending more time, being uncomfortable while in transport | Transport adapted services Training | Environmental Factors | e5 Services, systems and policies | e540 Transportation services, systems and policies | e 5408 Transportation services, systems and policies, other specified (transport adapted) |
| *S05:"And you have an autistic person screaming the whole time. I can't handle noises, and it was unbelievable, [while using transport adapted]"* | Stroke | Impact of noise while using services; limiting future participation | Transport adapted services | Environmental Factors | e5 Services, systems and policies | e540 Transportation services, systems and policies | e 5408 Transportation services, systems and policies, other specified (transport adapted) |
| *S04:"Exactly, but the thing about the para transit is yeah you have this para transit service when you call and they are good, if they hire the taxis, and or they put them on the training course"* | Stroke | Some services (transit) are beneficial as long as they have appropriate training | Transport adapted services Training | Environmental Factors | e5 Services, systems and policies | e540 Transportation services, systems and policies | e 5408 Transportation services, systems and policies, other specified (transport adapted) |
| *S05:"Well, um I stopped with the transport adapted about a year ago probably, I use Uber, taxis, and the bus once in a while"* | Stroke | Option for community transit helps with mobility (transport, uber, taxi); Choices can facilitate participation | Public transportation services | Environmental Factor | e5 Services, systems and policies | e540 Transportation services, systems and policies | e 5408 Transportation services, systems and policies, other specified (Public transportation) |
| *S05: "usually, I'll go to an appointment , like actually when I was coming here, I'd take the bus here and then I would take an Uber home"* | Stroke | Option for community transit helps with mobility (transport, uber, taxi); Choices can facilitate participation | Public transportation services | Environmental Factor | e5 Services, systems and policies | e540 Transportation services, systems and policies | e 5408 Transportation services, systems and policies, other specified (Public transportation) |
| *C07: "sometimes, when we see [the patient], we can ask for transport adapted early enough when they are off work, but in any case, there is a [long] delay"* | Clinician | Limited access to (transportation) services | Transport adapted services | Environmental Factors | e5 Services, systems and policies | e540 Transportation services, systems and policies | e 5408 Transportation services, systems and policies, other specified (transport adapted) |
| *C05: "the transport adapted is not easy to use for patients with too much cognitive impairment"* | Clinician | Transport adapted/ poor service for people with cognitive impairment | Transport adapted services | Environmental Factors | e5 Services, systems and policies | e540 Transportation services, systems and policies | e 5408 Transportation services, systems and policies, other specified (transport adapted) |
| *C05: "it's too difficult to manage, they're not able to call, they're not able to give all the information"* | Clinician | transport adapted/ poor service for people with cognitive impairment | Transport adapted services | Environmental Factors | e5 Services, systems and policies | e540 Transportation services, systems and policies | e 5408 Transportation services, systems and policies, other specified (transport adapted) |
| *C06: "[patient who are not assigned for transport adapted] have a problem to get an access* | Clinician | Accessibility to outpatient rehabilitation, transportation services | Transport adapted services | Environmental Factors | e5 Services, systems and policies | e540 Transportation services, systems and policies | e 5408 Transportation services, systems and policies, other |

| Quotes | Population | Formulated statements | Codes | ICF domain | 1st Level ICF classification | 2nd Level ICF category | 3rd or higher Level ICF category |
|---|---|---|---|---|---|---|---|
| *to outpatient services and have no way to get there"* | | | | | | | specified (transport adapted) |
| *C01: "[when] there is a snow storm, transport adapted becomes very difficult to use"* | Clinician | Weather, access to transport service (timing, location) | Transport adapted services<br>Weather | Environmental Factor | e1 Products and technology<br><br>e5 Services, systems and policies | e150 Design, construction and building products and technology of buildings for public use<br>e540 Transportation services, systems and policies | e 1500 Design, construction and building products and technology for entering and exiting buildings for public use<br>e 5408 Transportation services, systems and policies, other specified (transport adapted) |
| *C01: "the reality is just they often they cannot come"* | Clinician | Weather, access to transport service (timing, location) | Transport adapted services<br>Weather | Environmental Factors | e1 Products and technology<br><br>e5 Services, systems and policies | e150 Design, construction and building products and technology of buildings for public use<br>e540 Transportation services, systems and policies | e 1500 Design, construction and building products and technology for entering and exiting buildings for public use<br>e 5408 Transportation services, systems and policies, other specified (transport adapted) |
| *C04: "if there is a snow storm, just forget it [because] the transportation is very late or transport adapted doesn't park close to them"* | Clinician | Weather, access to transport service (timing, location) | Transport adapted services<br>Weather | Environmental Factors | e1 Products and technology<br><br>e5 Services, systems and policies | e150 Design, construction and building products and technology of buildings for public use<br>e540 Transportation services, systems and policies | e 1500 Design, construction and building products and technology for entering and exiting buildings for public use<br>e 5408 Transportation services, systems and policies, other specified (transport adapted) |
| *C03: "there is also a financial issue that limit client's mobility"* | Clinician | Limits of public (transportation) services and limits of out-of-pocket access due to limited funds | Public transportation services<br>Financial limitation | Environmental Factor | e5 Services, systems and policies<br>e1 Products and technology | e540 Transportation services, systems and policies<br>e165 Assets | e 5408 Transportation services, systems and policies, other specified (Public transportation)<br>e1650 Financial assets |
| *C06: "they don't have the money to pay for a taxi every time, and transport adapted takes 2 months before they are accepted"* | Clinician | Getting the transport adapted service/late | Transport adapted services | Environmental Factors | e5 Services, systems and policies | e540 Transportation services, systems and policies | e 5408 Transportation services, systems and policies, other |

| Quotes | Population | Formulated statements | Codes | ICF domain | 1st Level ICF classification | 2nd Level ICF category | 3rd or higher Level ICF category |
|---|---|---|---|---|---|---|---|
| | | | | | | | specified (transport adapted) |
| *S05: "people really do not give us those handicap seats, if you have a cane they do not care, oh my goodness, I guess I'm too young or something"* | Stroke | Lack of social norms for accessible seating on community transport | Public transportation services<br>Social norms | Environmental Factor | e5 Services, systems and policies<br>e1 Products and technology | e540 Transportation services, systems and policies<br>e150 Design, construction and building products and technology of buildings for public use | e 5408 Transportation services, systems and policies, other specified (Public transportation)<br>e 1500 Design, construction and building products and technology<br>for entering and exiting buildings for public use |
| *S06:"I was taking the bus to the general [hospital] I think and I had my cane, when we got there, the bus driver stopped, looked up and said, hold on I will help you cross the street"* | Stroke | Helpful social norms on community transport helps with mobility | Public transportation services<br>Social norms | Environmental Factor | e5 Services, systems and policies<br>e1 Products and technology | e540 Transportation services, systems and policies<br>e150 Design, construction and building products and technology of buildings for public use | e 5408 Transportation services, systems and policies, other specified (Public transportation)<br>e 1500 Design, construction and building products and technology<br>for entering and exiting buildings for public use |
| *S06:"that was exceptional, I was shocked, and I said, No, it's okay, relax. But that's one of how many?"* | Stroke | Helpful social norms on community transport helps with mobility | Public transportation services<br>Social norms | Environmental Factor | e5 Services, systems and policies<br>e1 Products and technology | e540 Transportation services, systems and policies<br>e150 Design, construction and building products and technology of buildings for public use | e 5408 Transportation services, systems and policies, other specified (Public transportation)<br>e 1500 Design, construction and building products and technology<br>for entering and exiting buildings for public use |
| *S05:"I've actually had a bus driver, that the bus was full, and nobody was giving up the handicap seats, and he actually stopped the bus and screamed at them, Get up and give in your seats. I was like wooo, thank you!"* | Stroke | Helpful social norms on community transport helps with mobility | Public transportation services<br>Social norms | Environmental Factor | e5 Services, systems and policies<br>e1 Products and technology | e540 Transportation services, systems and policies<br>e150 Design, construction and building products and technology of buildings for public use | e 5408 Transportation services, systems and policies, other specified (Public transportation)<br>e 1500 Design, construction and building products and technology<br>for entering and |

| Quotes | Population | Formulated statements | Codes | ICF domain | 1st Level ICF classification | 2nd Level ICF category | 3rd or higher Level ICF category |
|---|---|---|---|---|---|---|---|
| | | | | | | | exiting buildings for public use |
| *S02: "But if it is a handicap seat, they should always have to be blank, for disabled person"* | Stroke | Helpful social norms on community transport helps with mobility | Public transportation services<br>Social norms | Environmental Factor | e5 Services, systems and policies<br>e1 Products and technology | e540 Transportation services, systems and policies<br>e150 Design, construction and building products and technology of buildings for public use | e 5408 Transportation services, systems and policies, other specified (Public transportation)<br>e 1500 Design, construction and building products and technology<br>for entering and exiting buildings for public use |
| **Theme 5: Uncertainty about the provided services** | | | | | | | |
| *S04: "I asked myself if I would ever return normal and would my symptoms last for a life time. They recently told me at xxx that my physiotherapy sessions have ended"* | Stroke | Uncertainty of deficits and recovery limits mobility; lack of transition to community-based services | Uncertainty<br>Healthcare services | Not defined<br>Environmental Factor | e5 Services, systems and policies | e580 Health services, systems and policies | e5809 Health services, systems and policies, other unspecified |
| *T01:"nobody can give you like a timeline or you're never going to really be able to give a timeline, but more kind of an evolution symptoms, or maybe what to kind of expect based on your initial symptoms"* | TBI | Lack of timeline of recovery; education about individualized symptoms and recovery path | Uncertainty<br>Healthcare services | Not defined<br>Environmental Factor | e5 Services, systems and policies | e580 Health services, systems and policies | e5809 Health services, systems and policies, other unspecified |
| *T03:"So once they categorize them as symptoms [clinicians] they explained to me what I should expect as a result from those symptoms sort of explained how those things are going to affect me"* | TBI | Knowledge of symptoms; individualized therapy and recovery path was useful | Uncertainty<br>Healthcare services | Not defined<br>Environmental Factor | e5 Services, systems and policies | e580 Health services, systems and policies | e5809 Health services, systems and policies, other unspecified |
| *T03:"And how long it's going to take to recover and they didn't give me a timeframe, just that they said, oh, well, this is something that might take a while"* | TBI | Uncertainty about timeline; recovery path, navigating difficult | Uncertainty<br>Healthcare services | Not defined<br>Environmental Factor | e5 Services, systems and policies | e580 Health services, systems and policies | e5809 Health services, systems and policies, other unspecified |
| *T03:"And now, because there's been a break and physiotherapy at xxx have stopped. I don't really know what to do?"* | TBI | Transition of services with COVID; lack of services; Uncertainty | Uncertainty<br>Healthcare services | Not defined<br>Environmental Factor | e5 Services, systems and policies | e580 Health services, systems and policies | e5809 Health services, systems and policies, other unspecified |
| *T03:"But I just started my therapy when all this, when the COVID19 started so I really* | TBI | Transition of services with COVID; lack of services; Uncertainty | Uncertainty<br>Healthcare services | Not defined<br>Environmental Factor | e5 Services, systems and policies | e580 Health services, systems and policies | e5809 Health services, systems and policies, other unspecified |

| Quotes | Population | Formulated statements | Codes | ICF domain | 1st Level ICF classification | 2nd Level ICF category | 3rd or higher Level ICF category |
|---|---|---|---|---|---|---|---|
| *haven't made any progress and I have no idea how to make progress at this point"* | | | | | | | |
| *T03: "I'm mostly resolved from it, but there's still some little symptoms and you kind of wonder, is that going to last forever or if there are ways to kind of do right now, especially because of all COVID as we don't have any therapies really like accessible"* | TBI | Uncertainty about lingering symptoms; uncertainty about transition of services during COVID | Uncertainty Healthcare services | Not defined Environmental Factor | e5 Services, systems and policies | e580 Health services, systems and policies | e5809 Health services, systems and policies, other unspecified |
| *T03:"And apart from having all the symptoms, I have this vision problem, I have double vision. For which I still do not have my corrective lenses for it's been over a year and waiting for the glasses"* | TBI | Comorbidities of symptoms; Vision symptoms an issue; Wait times for vision services a problem | Uncertainty Healthcare services | Not defined Environmental Factor | e5 Services, systems and policies | e580 Health services, systems and policies | e5809 Health services, systems and policies, other unspecified |

C: clinician perspective; S: stroke perspective; T: traumatic brain injury perspective
